# MTM-6, a Phosphoinositide Phosphatase, is Required to Promote Synapse Formation in *Caenorhabditis elegans*

**DOI:** 10.1371/journal.pone.0114501

**Published:** 2014-12-05

**Authors:** Vivian R. Ericson, Kerri A. Spilker, Madina S. Tugizova, Kang Shen

**Affiliations:** Department of Biology, Howard Hughes Medical Institute, Stanford University, Stanford, California, United States of America; University of Texas Medical Branch, United States of America

## Abstract

Forming the proper number of synapses is crucial for normal neuronal development. We found that loss of function of the phosphoinositide phosphatase *mtm-6* results in a reduction in the number of synaptic puncta. The reduction in synapses is partially the result of MTM-6 regulation of the secretion of the Wnt ligand EGL-20 from cells in the tail and partially the result of neuronal action. MTM-6 shows relative specificity for EGL-20 over the other Wnt ligands. We suggest that the ability of MTM-6 to regulate EGL-20 secretion is a function of its expression pattern. We conclude that regulation of secretion of different Wnt ligands can use different components. Additionally, we present a novel neuronal function for MTM-6.

## Introduction

Construction of functional nervous systems depends on the ability of neurons to form precise connections. The process of forming functional synapses begins with proper axon guidance and ends with the formation of synaptic contacts on specific cellular domains. As part of this process, neurons must form and maintain the appropriate number of synapses with their partners. Synapse formation and elimination is dynamic throughout the life of the neuron [1]–[5]. An excess of connections is formed early in development, and then a subset of synapses is eliminated as the neural circuits matures [1]–[3]. Abnormal synapse number is found in several forms of mental retardation, and a reduced number of synapses is strongly correlated with cognitive impairment in Alzheimer's Disease [5], [6].

Much of the research on synapse number has focused on changes in the dendritic arbor and dendritic spines in the mammalian central nervous system using dissociated cultured neurons [1]–[3], [5]–[7]. In hippocampal cultures, the calcium influx that occurs during stimulus triggers dephosphorylation of the transcription factor myocyte enhancement factor 2 (MEF2) [8], [9]. In turn, MEF2 activity limits the number of dendritic spines that form [8]. Additionally, an RNAi screen in cultured hippocampal neurons gave a number of potential cues for factors affecting synapse number including the cadherins, the semaphorins, and the GTPase REM2 [10]. Yet, the heterogeneous cell population and the issue of separating changes in morphology from changes in number present difficulties in understanding the molecular mechanisms controlling the synapse number. Here, we investigated the molecular mechanisms that regulate synapse number in an *in vivo* cell system in a neuron that exhibits a stereotyped number of synapses. We demonstrate a role for the myotublarin lipid phosphatase MTM-6 in the maintenance of proper synapse number.

The myotublarins are a disease-related family of dual-specificity phosphatases that act on phosphoinositides [11]–[18]. The founding family member, MTM1, was discovered through genetic studies that revealed an association with X-linked myotubular myopathy [16], [18], [19]. Additionally, mutations in the family members MTMR2 and MTMR13 cause Charcot-Marie-Tooth Types 4B1 and B2 neuropathy, respectively [20]–[22]. Individuals with Charcot-Marie-Tooth neuropathy exhibit degeneration of the peripheral nerves and muscle weakness starting during early childhood [20]–[22]. Similar to the phosphoinositides they regulate, the myotubularins are reported to have a broad number of functions including regulation of endocytosis, phagosome maturation, regulation of actin structure, cell proliferation, and cell survival [11]–[15], [23]–[35]. While in humans there are 14 myotubularin family members, the *C. elegans* genome encodes only five myotubularin genes [11]–[15].

The myotubularins act specifically on phosphatidylinositol 3-phosphate [PI(3)P] and phosphatidylinositol (3,5)-bisphosphate [PI(3,5)P2] [11]–[15], [18]. Phosphoinositides are important signaling lipids that are involved in many aspects of cell biology including synapse formation and maintenance [13], [14]. Loss of *MTMR-2*, a homolog of *C. elegans mtm-1*, causes a reduction in the number of dendritic spines in the excitatory neurons of the rodent brain [36]. PTEN and PI3K, both regulators of phosphatidylinositol (3,4,5)-triphosphate [PI(3,4,5)P3], also have reported affects on synapse number in both rodent and *Drosophilia* [37]–[39]. Previously, *C. elegans mtm-6* was shown to regulate endocytosis through its action on PI(3)P in coelomocytes, which are cells that parallel the function of phagocytic immune cells in higher organisms [23], [24].

*mtm-6* is also important for neuronal migration and neuronal polarity as a result of its role in controlling Wnt secretion in *C. elegans* [30]. The Wnt family of signaling molecules has diverse functions in development and disease [40], [41]. In the development of the nervous system Wnt molecules show functions in neuronal migration, axon guidance, neuronal polarity, dendritic morphogenesis, the formation of synapses, and synaptic function [42]–[58]. We report that loss of *egl-20* has a similar reduction in synapse number to loss of *mtm-6*. Using genetics, we conclude that *mtm-6* is required to maintain proper synapse number and that it does through in part through regulating secretion of the Wnt ligand EGL-20. In addition to regulating *egl-20*, *mtm-6* has an *egl-20* independent, neuronal function for maintaining synapse number.

## Results

### Loss of the myotubularin phosphatase *mtm-6* causes a reduction of synapse number

**Figure 1:**
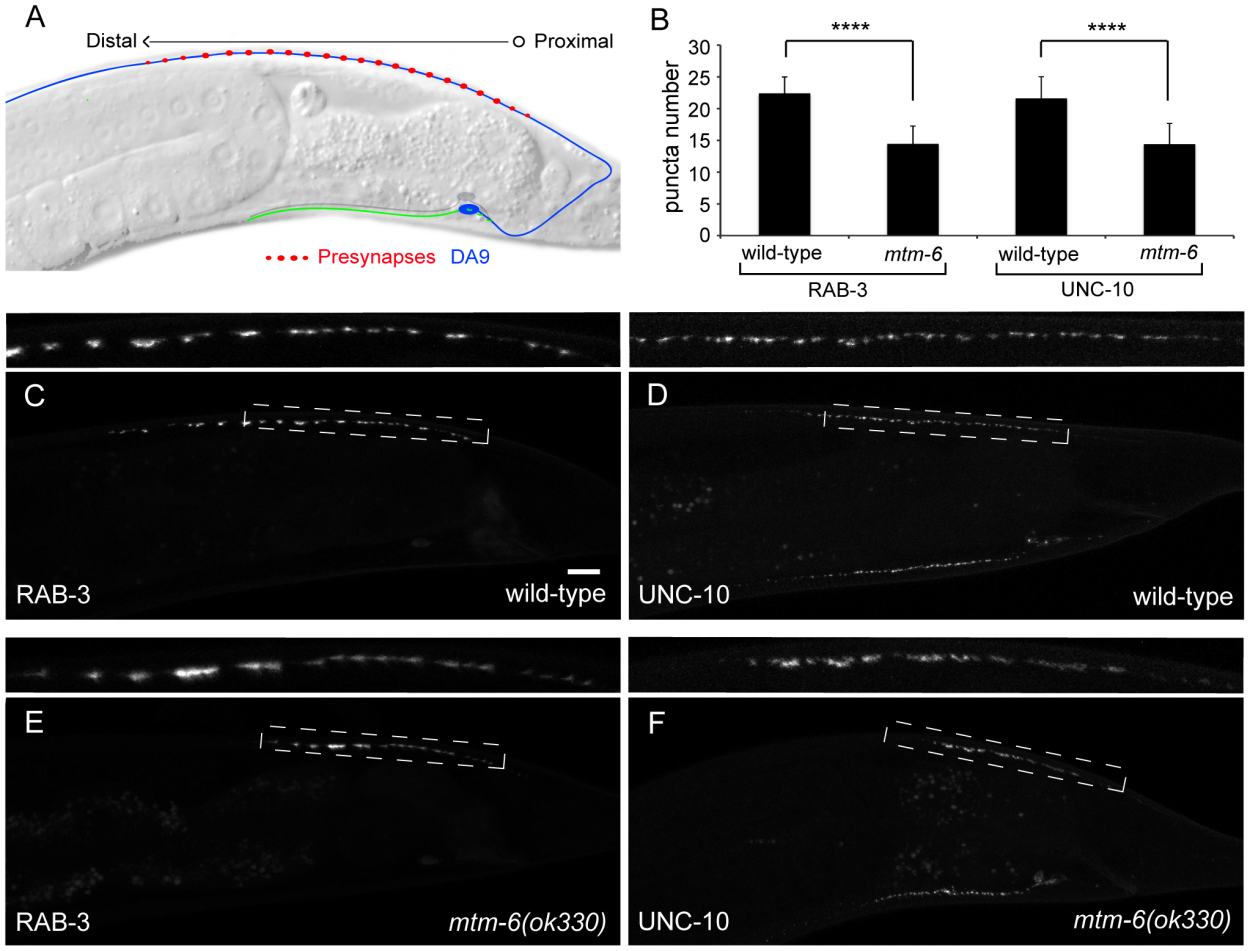
Loss of *mtm-6* causes a reduction in the number of DA9 presynaptic puncta. (A) Schematic of DA9. The dendrite of DA9 extends anterior from a cell body in the ventral side of the animal. The axon of DA9 extends towards the posterior before extending a commissure to the dorsal nerve cord, where the axon turns and continues towards the anterior of the animal. Synapses form with a stereotyped number and location on to muscle and Ventral D neurons. (B) Quantification of the number of puncta formed in wild-type and *mtm-6 (ok330)* animals using GFP::RAB-3 to mark presynapses and UNC-10::GFP as a marker for active zones (n>60, **** is p<0.0001). (C-F) *mtm-6 (ok330)* animals show a reduced number of dorsal synaptic puncta when compared to wild-type animals for the GFP::RAB-3 and UNC-10::GFP markers. (C) Wild-type; GFP::RAB-3. (D) Wild-type; UNC-10::GFP. (E) *mtm-6(ok330)*; GFP::RAB-3. (F) *mtm-6(ok330)*; UNC-10::GFP. The UNC-10::GFP puncta on the ventral side of the animal correspond to the VA12 motor neuron. The dashed boxes mark the regions that are enlarged for the insets above each window. Error bars are SD. Scale bar is 10 µm.

The *C. elegans* motor-neuron DA9 has a stereotyped synapse localization and number. The axon of DA9 extends posteriorly from a ventral cell body and reaches the dorsal nerve cord through a commissure. Within the dorsal nerve cord, the axon extends anteriorly and forms a string of en passant synapses in a specific region along the axon that begins approximately 25 µm anterior to the dorsal commissural turn in adult animals (Fig. 1A). The DA9 synapses can be labeled by cell-specific expression of GFP fused with synaptic vesicle or active zone proteins [56]. During the search for genes required for proper development of the DA9 synapses, we found that a mutant for a myotubularin phosphatase, *mtm-6(ok330)*, shows a reduction in synapse number (Fig. 1C-F). Using GFP::RAB-3 as a marker for synapses, we observed that in wild-type animals there are an average of 22.4 (+/−2.6) puncta within the dorsal cord. Both the number and the subcellular distribution pattern of the GFP::RAB-3 puncta closely agree with what was reported from serial electron microscopy reconstruction studies, suggesting that the RAB-3 marker represents true synapses. We found that the number of DA9 synapses is reduced to 14.4 (+/−2.8) puncta in *mtm-6* mutant animals (Fig. 1B and Table S1, n>60, SD, p<0.0001). The reduction in synapse number is also present when synapses are visualized with an active zone marker: quantifying GFP::UNC-10, the *C.elegan*'*s* RIM homolog, we observed that *mtm-6* mutants have an average of 14.4 (+/−3.3) puncta in comparison to the 21.6 (+/−3.4) puncta found in wild-type (Fig. 1B and Table S1, n>60, SD, p<0.0001). The *mtm-6(ok330)* allele is a 1,235 bp deletion that is predicted to cause a frameshift towards the end of the phosphatase that affects all four isoforms of the gene. Due to the temperature sensitive nature of some mutants used in the paper and the temperature sensitivity of the observed phenotypes (as marked by GFP::RAB-3 or GFP::UNC-10) all animals were maintained at 20°C for at least two generations prior to quantification.

To understand how the *mtm-6* mutant synapse number phenotype arises during development, we counted the number DA9 synapses during development in wild-type and *mtm-6* mutants. For these experiments, we used the *mig-13* promoter, which is active in DA9 during early larval stages, to drive expression of SNB-1/synaptobrevin::YFP. The DA9 neuron is born embryonically and forms synapses before hatching. After hatching, as the animals develop through four larval stages to adulthood, the number of synapses gradually increases as the body lengthens (Fig. S1). There is a small, but significant, difference in the number of SNB-1::YFP puncta at the L1 stage between wild-type and *mtm-6* mutants (Fig. S1 and Table S1, 2.35 puncta, n = 40, SD, p<0.001). However, there is no significant difference between wild-type and *mtm-6* animals at either the L2 or L3 stages. After the L3 stage the number of wild-type puncta continues to increase. However, in *mtm-6* mutants, the number of puncta appears to stall between L3 and young adulthood. While an average of 2.35 puncta are added between the young adults and adults with one row of eggs in the *mtm-6* mutant, this number is lower than the average 4.6 puncta added between wild-type young adults and 1-row adults (Fig. S1 and Table S1). This developmental study shows that the synapse number phenotype in the *mtm-6* mutant is not likely due to synapse elimination or maintenance defects, but instead represents a compromised ability to add synapses during development.

### The inactive phosphatase MTM-9 acts with MTM-6

**Figure 2:**
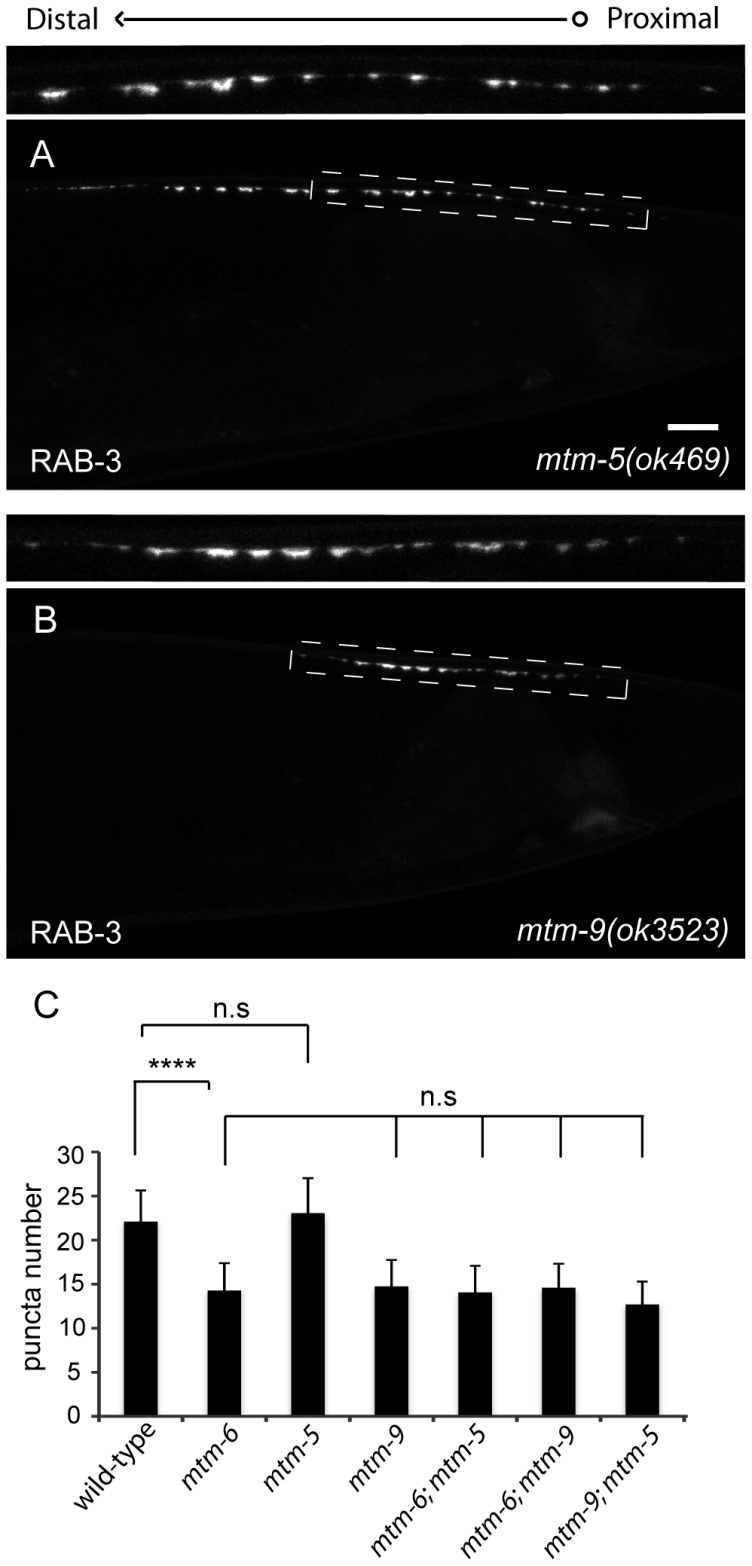
Loss of *mtm-9*, an inactive myotubularin, shows a reduced number of presynaptic puncta. (A) A mutant for the inactive myotubularin *mtm-5* has a similar number of GFP::RAB-3 puncta when compared to wild-type. (B) A mutant for the inactive myotubularin *mtm-9* has a reduced number of GFP::RAB-3 puncta, similar to the *mtm-6* mutant. (C) Quantification of single and double mutants for the inactive myotubularins (n>60, **** is p<0.0001, n.s. is not significant, and error bars are SD). The dashed boxes mark the regions that are enlarged for the insets above each window. Scale bar is 10 µm.

*mtm-6* encodes a myotubularin lipid phosphatase orthologous to human MTMR6-8, and in vivo it regulates phosphatidylinositol 3-phosphate levels. The myotubularin family has evolved to have both phosphatase active and phosphatase inactive members [15], [26], [59] . Phosphatase active myotubularins sometimes require the formation of heterodimers with phosphatase inactive myotubularins to function [15], [26], [59]. There is evidence that inactive members are necessary for proper localization and activity of their active counterparts [15], [26], [59]. *C. elegans* has two inactive myotubularins, *mtm-9* and *mtm-5*. Previous reports have shown that *mtm-9* is the partner for *mtm-6* [15], [26], [59]. Consistently, the *mtm-5(ok469)* null allele causes no significant reduction in the number of GFP::RAB-3 puncta (Fig. 2A, C and Table S1, 23.4+/−3.1, n>60, SD); whereas, a strain carrying the *mtm-9(ok3523)* null allele has a reduction in the number of GFP-RAB-3 puncta that is comparable to the *mtm-6* mutant (Fig. 2B, C and Table S1 15.1+/−2.6, n>60, SD).

To test if *mtm-6* and *mtm-9* function together, we examined the phenotypes in the double mutants. The *mtm-9 (ok3523)* allele is a large deletion and a null [30]. If *mtm-6* and *mtm-9* function in the same genetic pathway, the GFP::RAB-3 phenotype in the double mutants should not be more severe when compared to either of the single mutants. Indeed, *mtm-6; mtm-9* double mutants show 14.8 (+/−2.3) GFP::RAB-3 puncta, which is not significantly different from *mtm-6* or *mtm-9* alone (Fig. 2C and Table S1, n>60, SD). The double mutant between the two inactive phosphatases shows no significant decrease, which suggests it is unlikely *mtm-5* functions with *mtm-6* or a different myotubularin to reduce the number of synaptic puncta (Fig. 2C and Table S1, 14.1+/−3.0, n>60, SD).

### *mtm-6* functions both within the DA9 neuron and within cells in the tail to promote DA9 synapse formation

**Figure 3:**
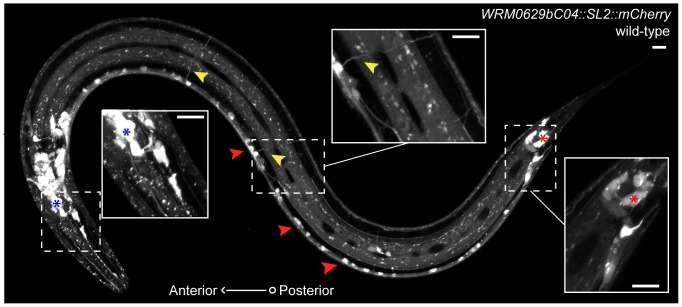
*mtm-6* is expressed in neurons. The expression pattern of *mtm-6* using the *WRM0629bC04* fosmid with the insertion of SL2::mCherry. Insets show head neurons (blue asterisk), commissures (yellow arrows), and non-neuronal cells (red asterisk) in the tail. Red arrows point to the ventral nerve cord neurons. Scale bars are 10 µm.

*mtm-6* is part of a large operon, which has made it challenging to observe its expression pattern directly. Previous studies on *mtm-6* used fragments of the operon to drive expression of fluorescent proteins, and they reported expression of the gene in the intestine [23]. Since the inactive myotubularin MTM-9 is known to act as a dimer with MTM-6, Silhankova et al. (2010) observed the *mtm-9* expression pattern and inferred that the *mtm-6* expression pattern would be similar. They found that *mtm-9* is expressed in the hypodermis, neurons, intestine, muscle, and epithelial cells, including the rectal epithelial cells known to secrete EGL-20. We tagged the fosmid containing the complete *mtm-6* operon with GFP::SL2::mCherry. This method revealed that *mtm-6* is expressed in many neurons throughout the worm, the pre-anal ganglion, hypodermal cells, the anal depressor muscle, and additional non-neuronal of cells in the tail (Fig. 3). The expression pattern is fairly constant through development.

The phenotype in the DA9 motor neuron and the expression pattern of *mtm-6* suggest the hypothesis that gene could act within neurons. Though some myotubularin family members affect muscle structure in humans, when we compared *mtm-6* mutant animals to wild-type we found that they exhibit normal muscle morphology (Fig. S2). As a result, the hypothesis that the neuronal phenotype of mtm-6 could arise from a defect in its post-synaptic partner is unlikely. An alternate hypothesis arises from the fact that MTM-6 is also reported to regulate Wnt secretion by influencing the recycling of the Wnt ligand carrier protein MIG-14/WIs [30]. Loss of *mtm-6* causes Wnt-like neuronal migration phenotypes in *C.elegans* [30]. The loss of *mtm-6* causes a visible reduction in the levels of MIG-14 in both *C.elegans* and *D. melanogaster* [30]. It is plausible that the reduction of GFP::RAB-3 puncta in the *mtm-6* mutant could be due to a defect in Wnt secretion.

**Figure 4:**
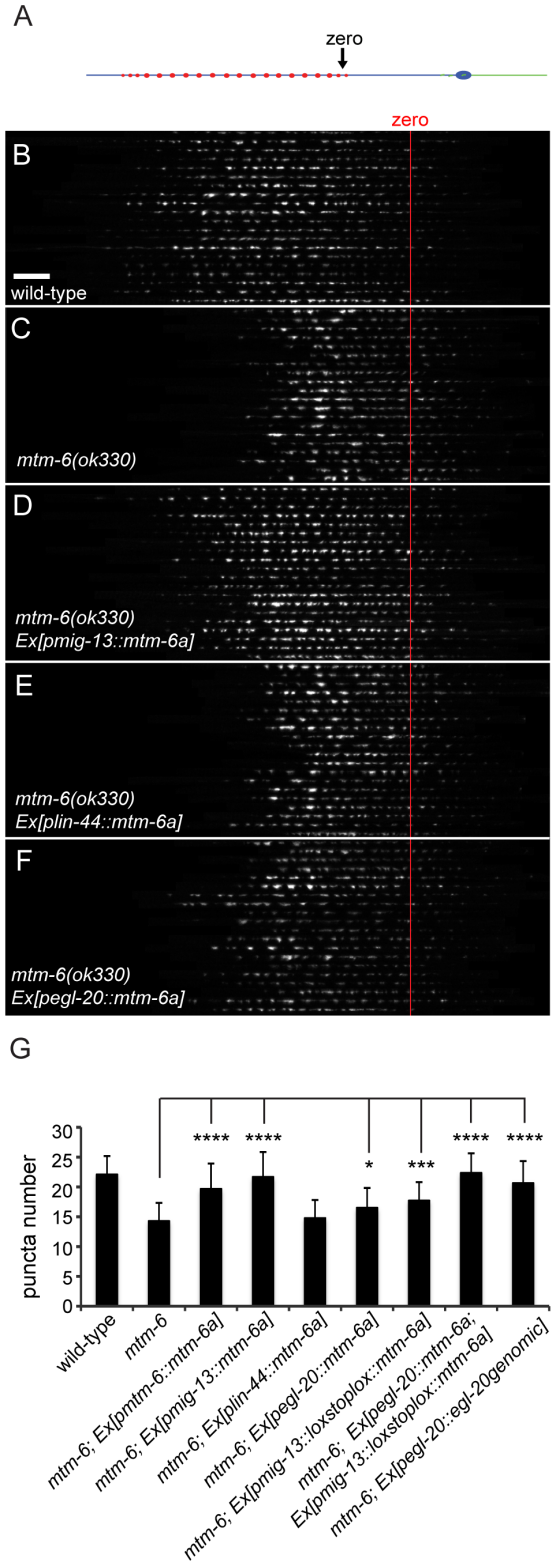
MTM-6 functions within the neuron and can be rescued by EGL-20 overexpression. A) Confocal images of DA9 axons were traced, straightened and aligned to produce an image montage. An image montage of the GFP::RAB-3 puncta from twenty animals for each strain was compiled for (B) wild-type, (C) *mtm-6 (ok330)*, (D) *mtm-6 (ok330); Ex[pmig-13::mtm-6a]*, (E) *mtm-6(ok330); Ex[plin-44::mtm-6a]*, (F) *mtm-6(ok330); Ex[pegl-20::mtm-6a]*. (G) Quantification of the GFP::RAB-3 puncta for *mtm-6(ok330)* and various rescue constructs. n>40, *** is p<0.001, n.s. is not significant, and error bars are SD.

To test where MTM-6 functions, we expressed *mtm-6* in various cell types in the *mtm-6* mutant animals. We observed significant rescue of the GFP::RAB-3 puncta number when we expressed genomic DNA containing *mtm-6* and its operon (Fig. 4G and Table S1, 19.7+/−4.3, p<0.0001, n>50, SD). We also created transgenic lines that express the cDNA of *mtm-6a*, one isoform of *mtm-6*, in the DA9 neuron (*mig-13* promoter), in LIN-44 secreting cells (*lin-44* promoter) and in cells expressing *egl-20* (*egl-20* promoter) (Fig. 4A-F). The *mig-13* promoter rescues the number of puncta in *mtm-6* mutants so that they are indistinguishable from wild-type levels (Fig. 4B-D, G and Table S1, 21.7+/−4.2, n>60, SD). This result suggests that *mtm-6* functions cell-autonomously in the DA9 neuron to regulate synapse number. Since the *mig-13* promoter is expressed in the VA12 neuron and a couple of tail cells in addition to DA9, we wanted to more stringently observe whether or not *mtm-6* functions in DA9. We placed a loxp::stop::loxp cassette between the *mig-13* promoter and *mtm-6a* so that expression of the cDNA would be suppressed in the absence of the cre recombinase. We expressed this construct in the background of a strain that contained a single copy insertion of cre driven by a promoter that is only expressed in the DA neurons. DA9 is the only cell that both the *mig-13* promoter and the DA specific promoter share, so the suppression of the expression of the *mtm-6a* cDNA is only removed within DA9. When we used this system to limit the expression of *mtm-6a* to DA9, we found that there is still significant rescue in animals containing the rescue construct with respect to the *mtm-6* mutant (Fig. 4G and Table S1, 17.7+/−3.1, p<0.0001, n>40, SD). The rescue is also significantly different from wild-type (p<0.0001), which implies that MTM-6 functions partially within the neuron, but might also have non-cell autonomous roles.

We observed no rescue of the *mtm-6* mutant with the *lin-44* promoter (Fig. 4E,G and Table S1, 14.8+/−3.0, n>60, SD). However, there is a slight but significant rescue of GFP::RAB-3 puncta number when expression of *mtm-6a* was driven with the *egl-20* promoter (Fig. 4F, G and Table S1, 16.7+/−2.9, p<0.011, n>60, SD). The modest rescue by the *egl-20* promoter and the fact that the *mig-13* promoter is more effective than the *mig-13*/cre-lox system suggests that the DA9 phenotype could be partially caused by MTM-6 regulating EGL-20 secretion. Consistent with this notion, overexpression of *egl-20* genomic DNA significantly rescues the *mtm-6* synapse phenotype (Fig. 4G, Fig. S6 and Table S1).

If the *mtm-6* phenotype is the result of a combined effect of *mtm-6* action within in the neuron and in the EGL-20 secreting cells, then coexpression of the *mtm-6a* cDNA using the *egl-20* promoter and the *mig-13/cre-lox* system should demonstrate improved rescue as compared with individual expression under the promoters. When we coexpressed *mtm-6a* from the two promoters we found that we achieved a rescue of the phenotype that is indistinguishable from wild-type (Fig. 4G and Table S1, 22.4+/−3.3).

Finally, we tested whether or not the phosphatase activity of MTM-6 is required to form the proper number of synapses using a previously characterized point mutation [24]. The introduction of the C335S mutation into the phosphatase domain of the *mtm-6a* construct prevents the rescue of the *mtm-6* mutant phenotype (Fig. S3 and Table S1, 16.95+/−3.1, n>40, SD). Furthermore, the C335S mutation causes no dominant negative effect in the wild-type background (Fig. S3 and Table S1). This suggests the phosphatase domain is required to maintain the proper number of synapses in DA9.

### MTM-6 functions partially through EGL-20 secretion to promote DA9 synapse formation

Our previous works show that two wnt gradients formed by LIN-44 and EGL-20 in the tail region of the worm inhibit synapse formation in the most posterior segment of DA9 axon [56], [60]. The synapse-free segments of the axon include the commissure and an “asynaptic” region in the dorsal DA9 axon [56], [60]. Crucially, Silhankova et al. (2010) observed that multiple Wnt processes are impacted by loss of *mtm-6*. Consistent with the hypothesis that the DA9 phenotype in *mtm-6* mutants is partially due to impaired Wnt secretion, we observed a subtle shift of synaptic material into the asynaptic region of the neuron with respect to wild-type (Fig. 4B-C). Therefore, we compared the phenotypes of the *mtm-6* mutant with those of different Wnt mutants.

**Figure 5:**
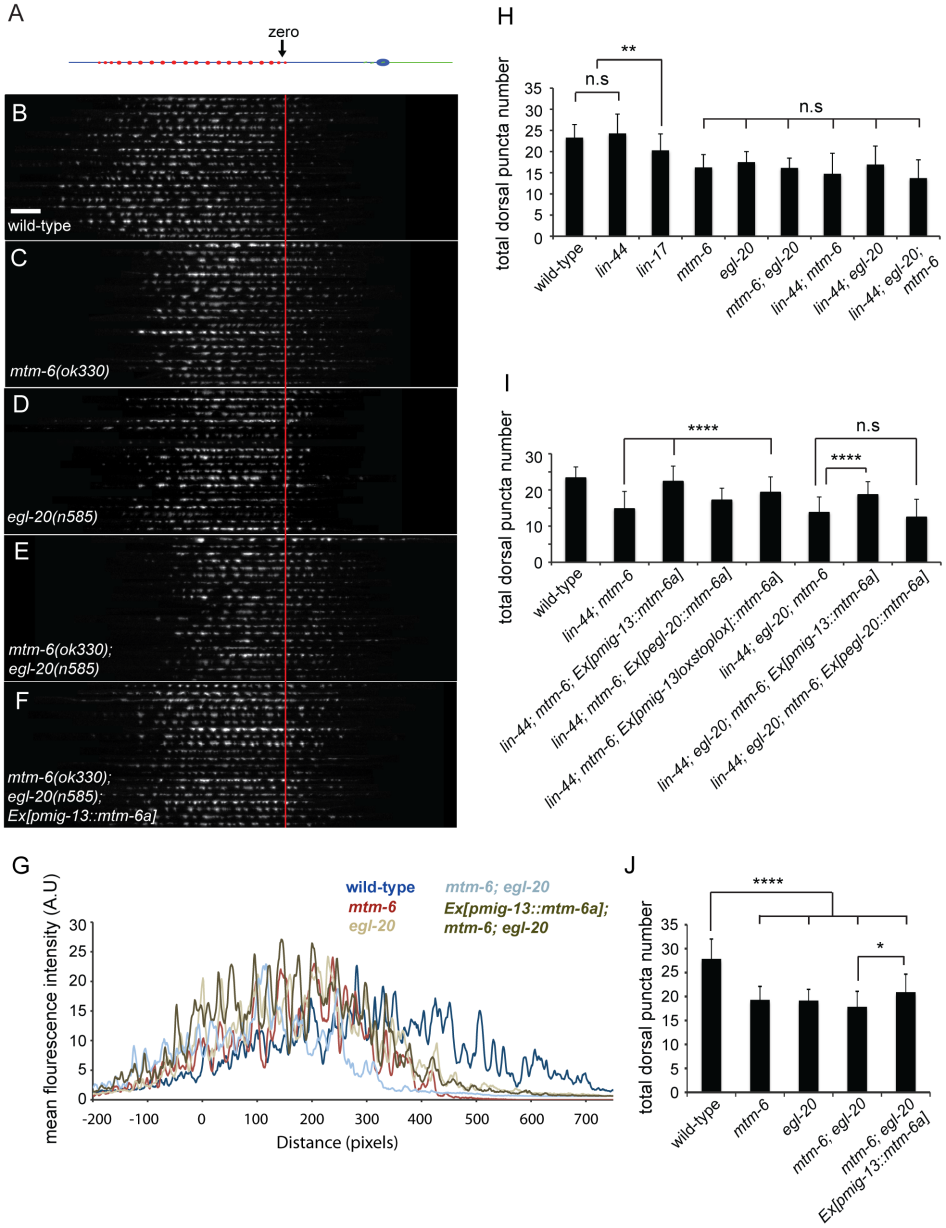
*egl-20* and *mtm-6* have similar reductions in synaptic puncta and act in a non-linear pathway. (A) Straightened DA9 neuron. Image montages of GFP::RAB-3 puncta for 20 animals were produced for (B) wild-type, (C) *mtm-6(ok330)*, (D) *egl-20(n585)*, (E) *mtm-6(ok330)*; *egl-20(n585)*, and *mtm-6(ok330)*; *egl-20(n585); Ex[Pmig-13::mtm-6a]*. (F) Linescans of mean fluorescence intensity with respect to distance of the aligned image montages. (H) Quantification of puncta number in single, double and triple mutants for different Wnt molecules. (I) Quantification of puncta number in the rescue of the different double and triple for different Wnt molecules. (J) Quantification of puncta number in the image montages (B-F) using ImageJ. For (H and I) n = 40, **** is p<0.0001, ** is p<0.01, * is p<0.05, n.s. is not significant, and error bars are SD. For (J) n = 20.

There are five Wnt ligand genes in the *C. elegans* genome: *lin-44*, *egl-20*, *cwn-1*, *cwn-2* and *mom-2*. We focused our attention on the first four as *mom-2* predominantly functions in endoderm specification [61]. To better understand the *mtm-6* phenotypes, we counted the total number of puncta in the dorsal region, the number of puncta in the dorsal asynaptic region and the number of puncta in the commissure (Fig. 5A). The quantification of these parameters allows for characterization of two different synaptic phenotypes. The first phenotype is the number of synapses. The second phenotype is the position of the synapses. The loss of inhibitory Wnt signaling causes a shift of synaptic material into the dorsal asynaptic region. The most severe Wnt phenotypes show an additional shift of material into the commissure.

As previously shown, *lin-44(n1792)* shows a marked shift of GFP::RAB-3 puncta into the dorsal asynaptic area as a single mutant, but no change in the total puncta number compared to wild-type (Fig. 5H, Fig. S4 and Table S1). While *cwn-1(ok546)* and *cwn-2(ok895)* have slight reductions of puncta number (Fig. S5A), only *egl-20(n585)* shows a reduction in puncta number that is comparable to the *mtm-6* mutant (Fig. 5C-D, H, Fig. S6 and Table S1, *egl-20(n585)* 17.5+/−2.5 compared with *mtm-6(ok330)* 16.5+/−3.1, n = 40, SD, p>0.9999). These results suggest that *mtm-6* might function through regulating EGL-20 secretion. To investigate whether *egl-20* and *mtm-6* could act in a single linear pathway or in parallel pathways, we observed the synaptic phenotypes in the double and triple mutants.

**Figure 6:**
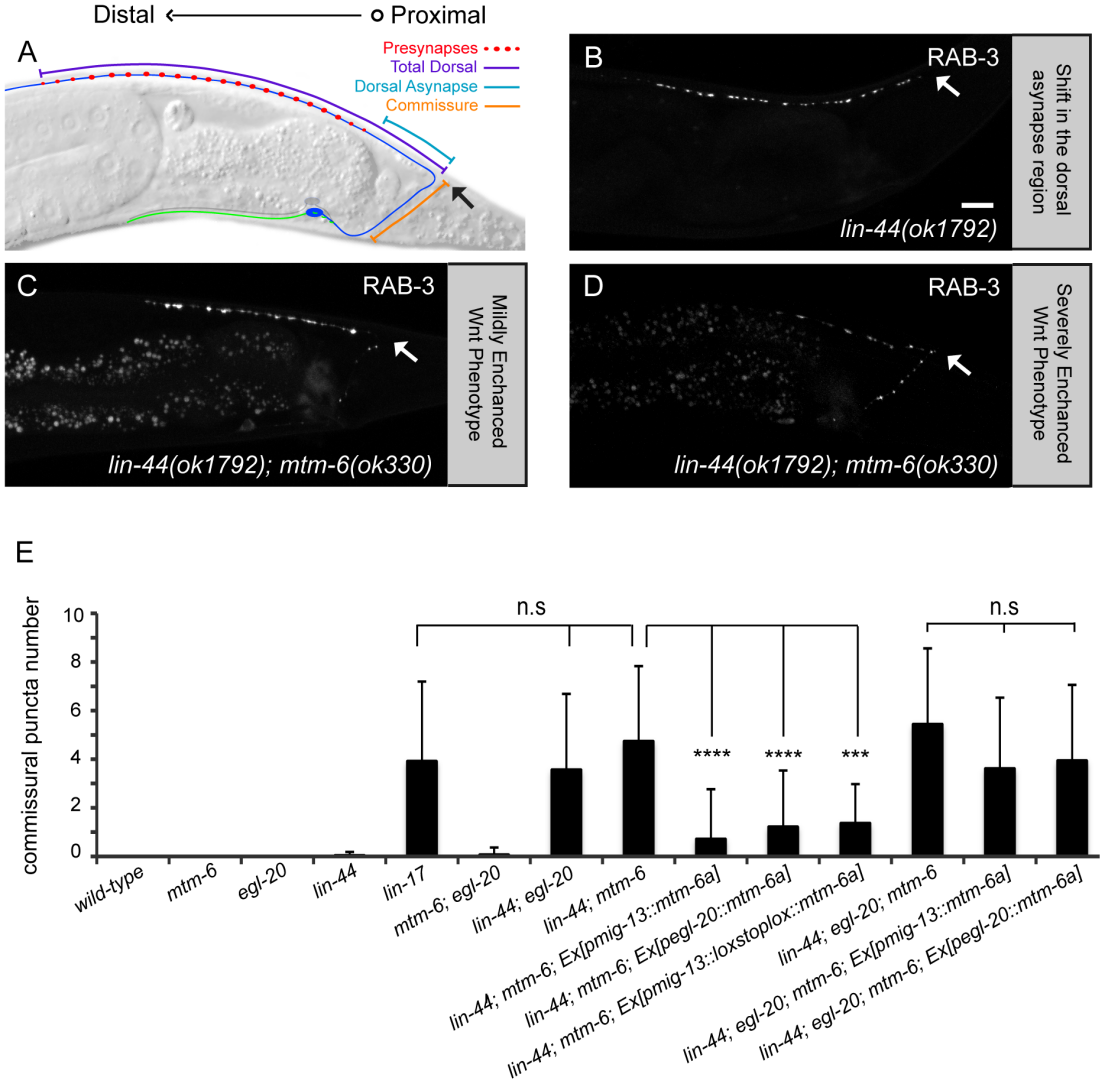
Loss of *mtm-6* enhances the Wnt phenotype in DA9 in an *egl-20* dependent manner. (A) Schematic of DA9. GFP::RAB-3 puncta were quantified in the commissural region, the dorsal asynaptic region and the total dorsal region. *lin-44(n1792)* shows a stereotyped shift of GFP::RAB-3 into the dorsal asynaptic region of DA9 (B). *lin-44(n1792); mtm-6(ok330)* animals have GFP::RAB-3 puncta in the commissure (C,D). (E) Quantification of the number of commissural puncta to measure the enhancement of the Wnt phenotype by *mtm-6* and to measure the cell-autonomous rescue experiments. n = 40, **** is p<0.0001, n.s is not significant, and error bars are SD. Arrows in (A-D) mark the DA9 dorsal commissure turn. Scale bar is 10 µm.

If MTM-6 primarily regulates EGL-20 secretion, then there should be no enhancement between the *egl-20* and *mtm-6* mutants. Indeed, we found that the *mtm-6*; *egl-20* double mutant shows no significant enhancement of the synaptic number phenotype (Fig. 5C-E, H and Table S1, 16.1+/−2.3, n = 40, SD, p>0.9998). In contrast, the *mtm-6*; *lin-44* double mutant shows enhanced phenotypes with puncta appearing in the commissure (Fig. 6C-E and Table S1, 3.6+/−3.1, n = 40, SD). From prior reports, the appearance of puncta in the commissure is typical of loss of the frizzled receptor *lin-17* or loss of the combination of *egl-20* and *lin-44* (Fig. 6B-D and Table S1) [56], [60]. As previously mentioned, it represents a more severe loss of inhibitory positional cues. The enhancement of the misolocalization phenotype in the *lin-44* background by *mtm-6* is not significantly different from *lin-17(n671)* or the *lin-44*; *egl-20* double mutant (Fig. 6E and Table S1). There is no enhancement of the *lin-44* mutant by either the *cwn-1* or *cwn-2* mutants, suggesting that *cwn-1* and *cwn-2* are not involved (Fig. S5 and Table S1). The fact that the *mtm-6* and *egl-20* mutants enhance the *lin-44* mutant in a similar manner supports the hypothesis that MTM-6 regulates EGL-20 secretion.

If *mtm-6* and *egl-20* function in a linear pathway, then the *mtm-6*; *egl-20*; *lin-44* triple mutant should not be enhanced as compared with the *lin-44; egl-20* or the *lin-44*; *mtm-6* double mutants. Consistent with this hypothesis, there is no significant increase in either the dorsal puncta number or commissural puncta number in the triple mutant (Fig. 5H, Fig. 6E, and Table S1). There is an increase in the incidence of defective axon guidance (data not shown). In the *lin-44; egl-20* or the *lin-44*; *mtm-6* double mutants, the incidence of defective axon guidance is around 9–10%. The *mtm-6*; *egl-20*; *lin-44* triple mutant has an incidence of 28.6% for defective axon guidance. We have excluded the animals with axon guidance defects when scoring synapse phenotypes. Together, these genetic interaction results regarding the synapse phenotypes are consistent with the notion that *mtm-6* and *egl-20* function in the same genetic pathway to prevent DA9 synapses from forming in the asynaptic region. Yet, these results do not explain the neuronal function of the gene or exclude the possibility that *mtm-6* could function in a parallel and non-linear pathway.

To further understand the cellular action of *egl-20* and *mtm-6*, we tested whether *mtm-6* is required in EGL-20 secreting cells with additional cell-autonomous rescue experiments. The initial rescue experiments of the *mtm-6* single mutant suggest that the *mtm-6* synapse number phenotype is partially dependent on *egl-20*, since both the *mig-13*/cre-lox system and the *egl-20* promoter partially rescue the phenotype, but can fully rescue the phenotype in combination (Fig. 4G). If this hypothesis is true, then we should see partial rescue of the synapse number phenotype in the *egl-20; mtm-6* and the *lin-44; egl-20; mtm-6* mutants when *mtm-6a* is expressed under the *mig-13* promoter. Indeed, we observed partial rescue of the reduced synaptic number in the absence of *egl-20* (Fig. 5E-G, I-J, and Table S1). Not only is rescue possible in the *mtm-6; egl-20* double mutant, but it is possible to rescue the *lin-44; egl-20; mtm-6* mutant by the *mig-13* promoter driving *mtm-6a* (Fig. 5I and Table S1, 18.7+/−3.6, n = 40, p<0.0001, not significantly different from wild-type, SD). In contrast, no rescue is seen in the background of the triple mutant when *mtm-6a* is expressed under the *egl-20* promoter. This result is consistent with two separate functions of MTM-6 in regulating the synapse number of DA9: an EGL-20 independent, cell-autonomous function of *mtm-6* in DA9, and an EGL-20 dependent function in the EGL-20 secreting cells. While these results elucidate the relationship of *mtm-6* and *egl-20* in the context of the synapse number phenotype, they do not indicate their interaction with respect to the positional phenotype.

To further understand the interaction between *mtm-6* and *egl-20* in regulating the location of synapses, we examined the number of ectopic puncta in the commissure in *mtm-6; lin-44* as well as *lin-44; egl-20; mtm-6* mutants. The commissural puncta phenotype in the *mtm-6; lin-44* double mutant is rescued when *mtm-6a* is expressed from the *egl-20* promoter, suggesting that *mtm-6* is required for the secretion of EGL-20 for the purpose of specifying the asynaptic region (Fig. 6F). There is also a reduction in the number of commissural puncta when *mtm-6a* expression is driven by the *mig-13* promoter or the *mig-13*/cre-system (Fig. 6E and Table S1). However, the ability to rescue the enhanced Wnt phenotype by driving *mtm-6a* expression with all promoters tested is lost in the *lin-44; egl-20; mtm-6* mutant (Fig. 6E and Table S1). The data suggests that expressing *mtm-6a* from either neurons or the EGL-20-expressing cells is sufficient to create enough EGL-20 to rescue the enhanced Wnt phenotype in the double. Yet, the failure to rescue the triple mutant ensures that this process is through *egl-20* and not a parallel pathway. The relation of *mtm-6* and *egl-20* with respect to the phenotype of commissural puncta GFP::RAB-3 is linear, which is contrast to the non-linear relation of *mtm-6* and *egl-20* for the number of GFP::RAB-3 puncta.

### The Relative Specificity of *mtm-6* for EGL-20 secretion

**Figure 7:**
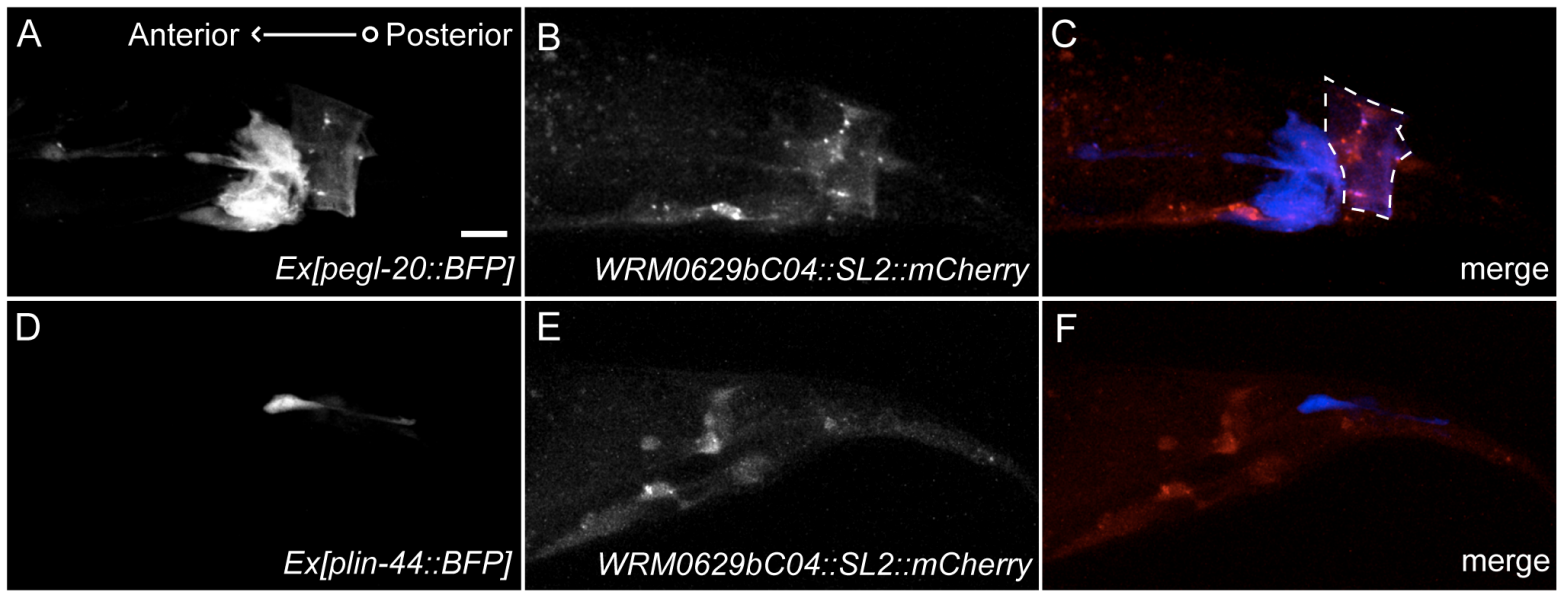
The expression pattern of *mtm-6* overlaps with the expression pattern of *egl-20*. When the expression pattern of *Ex[Pegl-20::BFP]* (A) is compared with the expression pattern of *Ex[WRM0629bC04::GFP::SL2::mCherry]* (B) there is overlap within the anal depressor muscle (C). When the expression pattern of *Ex[Plin-44::BFP]* (D) is compared with the expression pattern of *Ex[WRM0629bC04::GFP::SL2::mCh]* (E) there is no overlap in the merge (F). Scale bar is 10 µm. Dashed line highlights the region of the anal depressor muscle.

The genetic data and prior research by Silhankova et al. (2010) support the hypothesis that *mtm-6* acts predominantly on *egl-20* and not the other Wnt ligands. We hypothesized that the relative specificity of MTM-6 for EGL-20 secretion could result from the expression pattern of *mtm-6*. We observed whether or not there was overlap between the *mtm-6* tagged-fosmid expression pattern and the expression pattern of different Wnts. We found that there is overlap between the *mtm-6* and the *egl-20* expression patterns in the anal depressor muscle, but there is little to no overlap of the *mtm-6* expression pattern with the *lin-44* expression pattern (Fig. 7A-F). This implies that different Wnt ligands may rely on distinct components to their secretory paths based on where they are expressed.

## Discussion

Initial research on the myotubularin family focused on mutations in the myotubularin genes underlying X-linked myotubular myopathy and two Charcot-Marie-Tooth neuropathies. In the former case myotubularins act in muscle and in the second the myotubularins are required for myelination of neurons by Schwann cells [16], [21]. Yet, a growing body of research implies that myotubularins and the phosphoinositides they regulate also function in neurons. MTMR2 is expressed in peripheral nerves and is capable of affecting the number of dendritic spines in excitatory neurons [36], [62]. It is plausible that the neuropathies are caused in part by defects in neurons. Here we demonstrate that loss of *mtm-6* affects both the number of synaptic puncta and their location within the motor neuron DA9. We propose that *mtm-6* impacts synapses in part through its regulation of Wnt secretion and in part through a Wnt independent function within the neuron.

Genetic analysis shows that *mtm-6* largely acts on *egl-20* rather than *lin-44*, *cwn-1* or *cwn-2*. Loss of *mtm-6* strongly enhances the *lin-44* synapse mislocalization phenotype in a way that is very similar to the enhancement of *lin-44* by *egl-20* (Fig. 6). We observed no enhancement of *egl-20* by *mtm-6* with respect to the number of GFP::RAB-3 puncta (Fig. 5). Furthermore, neither *cwn-1* nor *cwn-2* enhances the *lin-44* synapse mislocalization (Fig. S5). The relative specificity of *mtm-6* for *egl-20* could arise from its expression pattern, which shows some overlap with *egl-20* but little to no overlap with *lin-44* (Fig. 7).

That loss of *mtm-6* seems to have a greater impact on *egl-20* rather than the other Wnt ligands is consistent with a previous study by Silhankova et al. (2010). In their report, several Wnt-mediated processes are observed to have varying dependencies on the different Wnt ligands. The impact of *mtm-6* on the different Wnt mediated processes is also variable. The *mtm-6* mutation has a dramatic effect on the QL cell migration, which is a process that is largely dependent on EGL-20 secretion [52], [55], [63]. Similarly, loss of *mtm-6* has a noticeable affect on HSN migration, a process that is known to be affected by loss of *egl-20* [55]. However, the *mtm-6* mutation has no affect on PLM polarization and only a small but reproducible affect on ALM polarization. PLM polarity is dependent on *lin-44*, *cwn-1* and *egl-20,* while ALM is dependent on *egl-20*, *cwn-1* and *cwn-2*. If *mtm-6* affected all Wnt secretion equally, then one would expect there to be a more marked affect on ALM and PLM polarization, as is seen by the more generally required Wnt secretion factor *mig-14/wntless* [55]. However, we acknowledge that interpreting the genetic interaction results both in our works and prior works is complicated with respect to the intricate series of secreted Wnt ligands in this system and that alternate models are possible.

How *mtm-6* is able to modulate synapse number within the neuron is unclear and could be of interest for future research. It is also unclear at this point how or if the synapse number phenotype of the *mtm-6* mutant relates to inhibitory Wnt positional cues. It is of interest is that *mtm-6* is expressed in many neurons. We observed a subtle backing defect in *mtm-6* worms, which assisted in genotyping *mtm-6* homozygous animals. When we observed other neurons we observed minor effects in AIY and PLM. The other active myotubularins, *mtm-1* and *mtm-3*, may act redundantly with *mtm-6* to prevent more widespread effects in the nervous system or to prevent more distinct movement defects. Myotubularin expression is also seen in the central nervous system and in peripheral nerves in mammalian systems. Myotubularins have been linked to Creutzfeldt-Jakob disease and epilepsy in genomic studies [64], [65]. *C. elegans* may provide a more genetically tractable system for understanding neuronal myotubularin function and shedding light on their roles in neuronal disease.

The reduced number of synaptic puncta in the *mtm-6* mutant led to the realization that *egl-20* mutants also have a reduced number of synaptic puncta. This novel observation was made possible by stringently maintaining consistent growth conditions across the different strains observed since the observed phenotypes were temperature sensitive as visualized by GFP::RAB-3 or GFP::UNC-10. That EGL-20 can modulate the number of puncta in DA9 highlights the Wnt family's diverse portfolio of functions in nervous system. Wnts are involved at nearly every step of neuronal circuit development, from the initial precursor migration to the fine-tuning of acetylcholine receptors at a synapse [42]–[58]. *egl-20* is known to have a strong effect in controlling the synaptic pattern of the DA9's neighbor, DA8 [60]. DA8 and DA9 form their synapses in adjacent but non-overlapping regions leading to tiled synaptic innervations [66]. *egl-20* inhibits synapse formation in DA8's asynaptic region in much the same way *lin-44* inhibits DA9's synapses to assist in creating the tiled synaptic domains [60]. *egl-20* may act directly on DA9, or it may be that DA9 reacts to the effect of *egl-20* on DA8.

*egl-20* and *mtm-6* appear to act in parallel paths with respect to controlling synapse number. EGL-20 secretion can influence the number of synapses in the *mtm-6* mutant, hinting that there may be overlap between the two genes further downstream of each pathway (Fig. 4G). The developmental data suggests that the *mtm-6* phenotype could be the result of inefficient addition of synapses in the process of animal growth (Fig. S1). The addition of synapses requires the biosynthesis of presynaptic material in the cell body, long-range transport of this material along the axon, and local aggregation of synaptic vesicle with active zone proteins. It seems plausible that the MTM-6 could influence vesicle trafficking through phospholipid modulation since phospholipids are implicated in several aspects of intracellular membrane trafficking. Future experiments will be required to determine the site of action for MTM-6 in synapse formation.

## Materials and Methods

### Strains and Genetics

Strains were maintained on *E. coli* OP50 seeded NGM plates and kept at 20°C. The following strains were received from the *Caenorhabditis* Genetics Center: VC161 *mtm-6(ok330)*, MT4051 *lin-44(n1792)I; him-5(e1490) V*, MT1306 *lin-17(n671)*, RB2541 *mtm-9(ok3523)*, VC263 *mtm-5(ok469)*, MT1215 *egl-20(n585) IV*, RB763 *cwn-1(ok546) II*, VC636 *cwn-2(ok895)IV* and, HS1675 *egl-20(n585) IV; cwn-2(ok895) IV, trIs30 (Phim-4p::MB::YFP; Phmr-1b::DsRed2; Punc-129nsp::DsRed2)*. N2 Bristol was used as the wild-type reference strain. The following primer sets were used for genotyping deletions: *mtm-6(ok330)* F1-tgcagtccaaactgtgaagc, *mtm-6(ok330)* R1-TAGGGCACGGGGTACTGTAG, *mtm-6(ok330)* R2-ttgacgcgcaaaatatctca, *mtm-5(ok469)* F1-ttgagcacagatcgatgagc, *mtm-5(ok469)* R1- gccaagacggaaacatgact, *mtm-5(ok469)* R2-TCGGAGATGTTGCAGAAAGA, *mtm-9(ok3523)* F1-tgctccgaacgaaaagtgacatcg, *mtm-9(ok3523)* R1-cgtttttcgggattttcgcgttgg, *cwn-1(ok546)* F1-gagcagccctgatattggaa, *cwn-1(ok546)* R1-gagataaaccgggaacgtca *cwn-1(ok546)* R2-TCGGCATTTGTACATGCAAT *cwn-2(ok895)* F1-tgcatccgtttaggtcaaca, *cwn-2(ok895)* R1-ggaacgcaggaaatgattgt, *cwn-2(ok895)* R2-TTCCATCGATGACCTGTGAA. *egl-20(n585)* substitution was genotyped using *egl-20(n585)*-L cctcattaccattcaactgatag *egl-20(n585)*-U cttacctctcaaatttgaacttattcttgc followed by a 3 hr digestion of the PCR product by HpyCH4V. The *lin-44(n1792)* substitution was genotyped using *lin-44(n1792)* NcoI-F GTGCGAATCGTTTGAGATTTCAGCCAT and *lin-44(n1792)* NcoI-R CATCTGGTTGTTACACGCACAATCG followed by a 3 hr digestion of the PCR product with NcoI. Digested PCR products for *egl-20(n585)* and *lin-44(n1792)* genotyping were run on a 2% agarose gel.

### Cloning and Constructs

Expression constructs utilized the pSM vector, which is a modified form of pPD49.26 [67] that contains extra cloning sites (S. McCarroll and C.I. Bargmann, communication with the Shen Lab). The following lines were generated as previously described: *wyIs85 (Pitr-1pB::GFP::rab-3), wyIs318 (Pmig13::unc-10::GFP; Pmig13::mCherry), wyEx5086 (Pmig-13::mtm-6a), wyEx5087 (Plin-44::mtm-6a)*, *wyEx5274 (Pegl-20::mtm-6a), wyEx5385 (WRM0629bC04::GFP::SL2::mCh), wyEx4667 (WRM0629bC04), wyEx5271 (Pmig-13::mtm-6a^C335S^), wyEx5273 (Pegl20::mtm-6a), wyEx6067 (Pegl-20::egl-20genomic), wyEx6259 (Phlh-1::egl-20genomic), wySi265 (Punc-4c::cre::Punc-122::mcherry::unc-119+), wyEx6471 (Pmig-13::loxstoplox::mtm-6a), wyEx6475 (Pmig-13::loxstoplox::mtm-6a), wyEx6286 (Pmig-13::loxstoplox::egl-20genomic), wyEx6469 (Pmig-13::loxstoplox::egl-20genomic)*, *wyEx6299 (Pegl-20::BFP)*, and *wyEx6260 (Plin-44::BFP)*. The co-injection markers used were either *Podr-1::GFP* or *dsRED* injected at 20 ng µl^−1^.

### Fluorescence Microscopy and Quantification

Images were taken using a Plan-Apochromat 40×1.3 objective on a Zeiss LSM710 confocal microscope. Worms were immobilized using a mixture of 5 mM Levamisole (Sigma-Aldrich) and 500 mM 2,3-butanedione monoxime (Sigma-Aldrich) on a 2% agarose pad. Image montages were created and analyzed following the methods previously described with one modification [56]. The images were aligned along anteroposterior axis using the region where the axon passes above the cell body and the gut rather than using DA9 commissure as previously reported.

Muscle arms were imaged by soaking worms in 3 mM muscimol for a 5 minutes and then transferring the worms on to a 5% agarose pad. The worms were rolled using the coverslip so that some of worms were dorsal side up.

Synapses were counted using a Zeiss AxioSkop or AxioVivison epifluorescent microscope with a mercury or X-cite light source. Worms were immobilized with 1 mM Levamisole on a 2% agarose pad. For each strain, n>40. Error bars represent Standard Deviation. Statistical analyses were performed with a one-way ANOVA test followed by Tukey's multiple comparis on test.

## Supporting Information

Figure S1 **The** ***mtm-6*** **phenotype is present at multiple stages of development.** Puncta number for SNB-1::YFP were counted for wild-type and *mtm-6 (ok330)* animals at L1-L4, young adult, 1-row adult and 2-row adult. n = 40, *** is p<0.001, **** is p<0.0001, n.s. is not significant, and error bars are SD.
(TIF)

Figure S2 ***mtm-6*** **mutant animals demonstrate largely normal muscle structure.** Muscle arms and muscle structure appear grossly normal when *mtm-6* mutants (B) are compared with wild-type animals (A).
(TIF)

Figure S3 **MTM-6 requires its phosphatase domain to maintain proper synapse number.** A construct of *mtm-6a* containing a point mutation in the phosphatase domain failed to rescue the *mtm-6* mutant. The construct had no affect on wild-type synapses. n>40, ****p<0.0001, n.s is not significant, and error bars are SD.
(TIF)

Figure S4 ***mtm-6*** **and** ***mtm-6; egl-20*** **have a subtle asynaptic phenotype.** Quantification of the aysynaptic shift phenotype in *mtm-6* and *mtm-6; egl-20* mutants compared to other Wnt mutants. n = 40, * is p<0.05, **** is p<0.0001, n.s is not significant, and error bars are SD.
(TIF)

Figure S5 ***cwn-1*** **and** ***cwn-2*** **mutants do not have the same pattern of Wnt enhancement as** ***mtm-6*** **.** Quantification and rescue data for different wnt mutants with *cwn-1(ok546)* (A and C) and *cwn-2(ok895)* (B and D). GFP::RAB-3 puncta were counted in the dorsal region (A and B) and the commissural region (C and D). n = 40,* is p<0.05, *** is p<0.001, **** is p<0.0001, n.s. is not significant, and error bars are SD.
(TIF)

Figure S6 **Egl-20 is not effectively rescued from neurons.** Image montages of confocal images of GFP::RAB-3 puncta were collected for (A) wild-type, (B) *egl-20(n585)*, (C) *egl-20(n585); Ex[pegl-20::egl-20]*. (D) Quantification of rescue for the total dorsal puncta phenotype of *egl-20(n585)* and *lin-44(n1792); egl-20(n585)*. (E) Quantification of rescue for the commissural phenotype of *egl-20(n585)* and *lin-44(n1792); egl-20(n585)*. n = 40,* is p<0.01, *** is p<0.001, **** is p<0.0001, n.s. is not significant, and error bars are SD.
(TIF)

Table S1 **Presentation of the raw data for the paper.** Mutants are represented within columns. Each row within a column represents a number for a single animal. The data is presented by figure and by region quantified.
(XLSX)

## References

[1] Huttenlocher PR (1979) Synaptic density in human frontal cortex-developmental changes and effects of ageing. Brain Res 163: 195–205. Available at: http://www.sciencedirect.com/science/article/pii/0006899379903494. Accessed January 9, 2014. 427544 10.1016/0006-8993(79)90349-4

[2] Huttenlocher PR (1990) Morphometric study of human cerebral cortex development. Neuropsychologia 28:517–527. 2203993 10.1016/0028-3932(90)90031-i

[3] Cohen S, Greenberg ME (2008) Communication between the synapse and the nucleus in neuronal development, plasticity, and disease. Annu Rev Cell Dev Biol 24: 183–209. Available at: http://www.pubmedcentral.nih.gov/articlerender.fcgi?artid=2709812&tool=pmcentrez&rendertype=abstract. Accessed December 22, 2013. 18616423 10.1146/annurev.cellbio.24.110707.175235 PMC2709812

[4] Bailey CH, Chen M (1988) Long-term memory in Aplysia modulates the total number of varicosities of single identified sensory neurons. Proc Natl Acad Sci U S A 85: 2373–7. Available at: http://www.pubmedcentral.nih.gov/articlerender.fcgi?artid=279995&tool=pmcentrez&rendertype=abstract. 3353385 10.1073/pnas.85.7.2373 PMC279995

[5] Honer W (2003) Pathology of presynaptic proteins in Alzheimer's disease: more than simple loss of terminals. Neurobiol Aging 24: 1047–1062. Available at: http://linkinghub.elsevier.com/retrieve/pii/S0197458003001763. Accessed January 7, 2014. 14643376 10.1016/j.neurobiolaging.2003.04.005

[6] Terry RD, Masliah E, Salmon DP (1991) Physical basis of cognitive alterations in Alzheimer's disease: synapse loss is the major correlate of cognitive impairment. Ann Neurol 30: 572–80. Available at: http://www.ncbi.nlm.nih.gov/pubmed/1789684. 1789684 10.1002/ana.410300410

[7] Lüthi A, Schwyzer L, Mateos JM, Gähwiler BH, McKinney RA (2001) NMDA receptor activation limits the number of synaptic connections during hippocampal development. Nat Neurosci 4: 1102–7. Available at: http://www.ncbi.nlm.nih.gov/pubmed/11687815. Accessed January 8, 2014. 11687815 10.1038/nn744

[8] Flavell SW, Cowan CW, Kim T-K, et al (2006) Activity-dependent regulation of MEF2 transcription factors suppresses excitatory synapse number. Science 311: 1008–12. Available at: http://www.ncbi.nlm.nih.gov/pubmed/16484497. Accessed December 18, 2013. 16484497 10.1126/science.1122511

[9] Shalizi A, Gaudillière B, Yuan Z (2006) A calcium-regulated MEF2 sumoylation switch controls postsynaptic differentiation. Science 311: 1012–7. Available at: http://www.ncbi.nlm.nih.gov/pubmed/16484498. Accessed February 7, 2014. 16484498 10.1126/science.1122513

[10] Paradis S, Harrar DB, Lin Y (2007) An RNAi-based approach identifies molecules required for glutamatergic and GABAergic synapse development. Neuron 53: 217–32. Available at: http://www.sciencedirect.com/science/article/pii/S0896627306009974. Accessed February 12, 2014. 17224404 10.1016/j.neuron.2006.12.012 PMC1950560

[11] Wishart MJ, Dixon JE (2002) PTEN and myotubularin phosphatases: from 3-phosphoinositide dephosphorylation to disease. Trends Cell Biol 12: 579–85. Available at: http://www.ncbi.nlm.nih.gov/pubmed/12495846. 12495846 10.1016/s0962-8924(02)02412-1

[12] Laporte J, Bedez F, Bolino A, Mandel JL (2003) Myotubularins, a large disease-associated family of cooperating catalytically active and inactive phosphoinositides phosphatases. Hum Mol Genet 12 Spec No (2): R285–92. Available at: http://www.ncbi.nlm.nih.gov/pubmed/12925573. Accessed January 8, 2014. 12925573 10.1093/hmg/ddg273

[13] Di Paolo G, De Camilli P (2006) Phosphoinositides in cell regulation and membrane dynamics. Nature 443: 651–7. Available at: http://www.ncbi.nlm.nih.gov/pubmed/17035995. Accessed December 12, 2013. 17035995 10.1038/nature05185

[14] Robinson FL, Dixon JE (2006) Myotubularin phosphatases: policing 3-phosphoinositides. Trends Cell Biol 16: 403–12. Available at: http://www.ncbi.nlm.nih.gov/pubmed/16828287. Accessed January 8, 2014. 16828287 10.1016/j.tcb.2006.06.001

[15] Hnia K, Vaccari I, Bolino A, Laporte J (2012) Myotubularin phosphoinositide phosphatases: cellular functions and disease pathophysiology. Trends Mol Med 18: 317–27. Available at: http://www.ncbi.nlm.nih.gov/pubmed/22578719. Accessed January 8, 2014. 22578719 10.1016/j.molmed.2012.04.004

[16] Laporte J, Hu LJ, Kretz C (1996) A gene mutated in X-linked myotubular myopathy defines a new putative tyrosine phosphatase family conserved in yeast. Nat Genet 13: 175–82. Available at: http://www.nature.com/ng/journal/v13/n2/pdf/ng0696-175.pdf. Accessed January 9, 2014. 8640223 10.1038/ng0696-175

[17] Laporte J, Blondeau F, Buj-Bello A (1998) Characterization of the myotubularin dual specificity phosphatase gene family from yeast to human. Hum Mol Genet 7: 1703–12. Available at: http://www.ncbi.nlm.nih.gov/pubmed/9736772. 9736772 10.1093/hmg/7.11.1703

[18] Blondeau F, Laporte J, Bodin S, Superti-Furga G, Payrastre B, et al (2000) Myotubularin, a phosphatase deficient in myotubular myopathy, acts on phosphatidylinositol 3-kinase and phosphatidylinositol 3-phosphate pathway. Hum Mol Genet 9: 2223–9. Available at: http://www.ncbi.nlm.nih.gov/pubmed/11001925. 11001925 10.1093/oxfordjournals.hmg.a018913

[19] Taylor GS, Maehama T, Dixon JE (2000) Myotubularin, a protein tyrosine phosphatase mutated in myotubular myopathy, dephosphorylates the lipid second messenger, phosphatidylinositol 3-phosphate. Proc Natl Acad Sci U S A 97: 8910–5. Available at: http://www.pubmedcentral.nih.gov/articlerender.fcgi?artid=16795&tool=pmcentrez&rendertype=abstract. 10900271 10.1073/pnas.160255697 PMC16795

[20] Mersiyanova IV, Perepelov AV, Polyakov AV, Sitnikov VF, Dadali EL, et al (2000) A new variant of Charcot-Marie-Tooth disease type 2 is probably the result of a mutation in the neurofilament-light gene. Am J Hum Genet 67: 37–46. Available at: http://www.pubmedcentral.nih.gov/articlerender.fcgi?artid=1287099&tool=pmcentrez&rendertype=abstract. 10841809 10.1086/302962 PMC1287099

[21] Bolis A, Coviello S, Bussini S, Dina G, Pardini C, et al (2005) Loss of Mtmr2 phosphatase in Schwann cells but not in motor neurons causes Charcot-Marie-Tooth type 4B1 neuropathy with myelin outfoldings. J Neurosci 25: 8567–77. Available at: http://www.ncbi.nlm.nih.gov/pubmed/16162938. Accessed January 8, 2014. 16162938 10.1523/JNEUROSCI.2493-05.2005 PMC6725661

[22] Robinson FL, Niesman IR, Beiswenger KK, Dixon JE (2008) Loss of the inactive myotubularin-related phosphatase Mtmr13 leads to a Charcot-Marie-Tooth 4B2-like peripheral neuropathy in mice. Proc Natl Acad Sci U S A 105: 4916–21. Available at: http://www.pubmedcentral.nih.gov/articlerender.fcgi?artid=2290800&tool=pmcentrez&rendertype=abstract. 18349142 10.1073/pnas.0800742105 PMC2290800

[23] Xue Y, Fares H, Grant B, Li Z, Rose AM, et al (2003) Genetic analysis of the myotubularin family of phosphatases in Caenorhabditis elegans. J Biol Chem 278: 34380–6. Available at: http://www.ncbi.nlm.nih.gov/pubmed/12788949. Accessed January 7, 2014. 12788949 10.1074/jbc.M303259200

[24] Dang H, Li Z, Skolnik EY, Fares H (2004) Disease-related Myotubularins Function in Endocytic Traffic in Caenorhabditis elegans. Mol Biol Cell 15:189–196. 14565969 10.1091/mbc.E03-08-0605 PMC307539

[25] Tsujita K, Itoh T, Ijuin T, Yamamoto A, Shisheva A, et al (2004) Myotubularin regulates the function of the late endosome through the gram domain-phosphatidylinositol 3,5-bisphosphate interaction. J Biol Chem 279: 13817–24. Available at: http://www.ncbi.nlm.nih.gov/pubmed/14722070. Accessed December 24, 2013. 14722070 10.1074/jbc.M312294200

[26] Lorenzo O, Urbé S, Clague MJ (2006) Systematic analysis of myotubularins: heteromeric interactions, subcellular localisation and endosome related functions. J Cell Sci 19: 2953–9. Available at: http://www.ncbi.nlm.nih.gov/pubmed/16787938. Accessed January 8, 2014. 10.1242/jcs.03040 16787938

[27] Zou J, Chang S-C, Marjanovic J, Majerus PW (2009) MTMR9 increases MTMR6 enzyme activity, stability, and role in apoptosis. J Biol Chem 284: 2064–71. Available at: http://www.pubmedcentral.nih.gov/articlerender.fcgi?artid=2629094&tool=pmcentrez&rendertype=abstract. Accessed January 8, 2014. 19038970 10.1074/jbc.M804292200 PMC2629094

[28] Taguchi-Atarashi N, Hamasaki M, Matsunaga K, Omori H, Ktistakis NT, et al (2010) Modulation of local PtdIns3P levels by the PI phosphatase MTMR3 regulates constitutive autophagy. Traffic 11: 468–78. Available at: http://www.ncbi.nlm.nih.gov/pubmed/20059746. Accessed January 8, 2014. 20059746 10.1111/j.1600-0854.2010.01034.x

[29] Razidlo GL, Katafiasz D, Taylor GS (2011) Myotubularin regulates Akt-dependent survival signaling via phosphatidylinositol 3-phosphate. J Biol Chem 286: 20005–19. Available at: http://www.pubmedcentral.nih.gov/articlerender.fcgi?artid=3103374&tool=pmcentrez&rendertype=abstract. Accessed December 18, 2013. 21478156 10.1074/jbc.M110.197749 PMC3103374

[30] Silhankova M, Port F, Harterink M, Basler K, Korswagen HC (2010) Wnt signalling requires MTM-6 and MTM-9 myotubularin lipid-phosphatase function in Wnt-producing cells. EMBO J 29: 4094–105. Available at: http://www.pubmedcentral.nih.gov/articlerender.fcgi?artid=3018790&tool=pmcentrez&rendertype=abstract. Accessed January 6, 2014. 21076391 10.1038/emboj.2010.278 PMC3018790

[31] Zou W, Lu Q, Zhao D, Li W, Mapes J, et al (2009) Caenorhabditis elegans myotubularin MTM-1 negatively regulates the engulfment of apoptotic cells. PLoS Genet 5: e1000679 Available at: http://www.pubmedcentral.nih.gov/articlerender.fcgi?artid=2751444&tool=pmcentrez&rendertype=abstract. Accessed January 9, 2014. 19816564 10.1371/journal.pgen.1000679 PMC2751444

[32] Mei J, Li Z, Gui JF (2009) Cooperation of Mtmr8 with PI3K regulates actin filament modeling and muscle development in zebrafish. PLoS One 4: e4979 Available at: http://www.pubmedcentral.nih.gov/articlerender.fcgi?artid=2656612&tool=pmcentrez&rendertype=abstract. Accessed January 9, 2014. 19325702 10.1371/journal.pone.0004979 PMC2656612

[33] Neukomm LJ, Nicot A-S, Kinchen JM, Almendinger J, Pinto SM, et al (2011) The phosphoinositide phosphatase MTM-1 regulates apoptotic cell corpse clearance through CED-5-CED-12 in C. elegans. Development 138: 2003–14. Available at: http://www.ncbi.nlm.nih.gov/pubmed/21490059. Accessed January 9, 2014. 21490059 10.1242/dev.060012

[34] Saarikangas J, Zhao H, Lappalainen P (2010) Regulation of the Actin Cytoskeleton-Plasma Membrane Interplay by Phosphoinositides. Physiol Rev 90:259–289. 20086078 10.1152/physrev.00036.2009

[35] Velichkova M, Juan J, Kadandale P, Jean S, Ribeiro I, et al (2010) Drosophila Mtm and class II PI3K coregulate a PI(3)P pool with cortical and endolysosomal functions. J Cell Biol 190: 407–25. Available at: http://www.pubmedcentral.nih.gov/articlerender.fcgi?artid=2922644&tool=pmcentrez&rendertype=abstract. Accessed January 9, 2014. 20696708 10.1083/jcb.200911020 PMC2922644

[36] Lee HW, Kim Y, Han K, Kim H, Kim E (2010) The phosphoinositide 3-phosphatase MTMR2 interacts with PSD-95 and maintains excitatory synapses by modulating endosomal traffic. J Neurosci 30: 5508–18. Available at: http://www.ncbi.nlm.nih.gov/pubmed/20410104. Accessed December 19, 2013. 20410104 10.1523/JNEUROSCI.4283-09.2010 PMC6632333

[37] Acebes A, Morales M (2012) At a PI3K crossroads: lessons from flies and rodents. Rev Neurosci 23:29–37. 22718611 10.1515/rns.2011.057

[38] Fraser MM, Bayazitov IT, Zakharenko SS, Baker SJ (2008) Phosphatase and tensin homolog, deleted on chromosome 10 deficiency in brain causes defects in synaptic structure, transmission and plasticity, and myelination abnormalities. Neuroscience 151: 476–88. Available at: http://www.pubmedcentral.nih.gov/articlerender.fcgi?artid=2278004&tool=pmcentrez&rendertype=abstract. Accessed January 8, 2014. 18082964 10.1016/j.neuroscience.2007.10.048 PMC2278004

[39] Jordán-Álvarez S, Fouquet W, Sigrist SJ, Acebes A (2012) Presynaptic PI3K activity triggers the formation of glutamate receptors at neuromuscular terminals of Drosophila. J Cell Sci 125: 3621–9. Available at: http://www.ncbi.nlm.nih.gov/pubmed/22505608. Accessed January 8, 2014. 22505608 10.1242/jcs.102806

[40] Logan CY, Nusse R (2004) The Wnt signaling pathway in development and disease. Annu Rev Cell Dev Biol 20: 781–810. Available at: http://www.ncbi.nlm.nih.gov/pubmed/15473860. Accessed December 12, 2013. 15473860 10.1146/annurev.cellbio.20.010403.113126

[41] Eisenmann DM (2005) Wnt signaling. WormBook. 25: 1–17. Available at: http://www.ncbi.nlm.nih.gov/pubmed/18050402. Accessed December 18, 2013. 10.1895/wormbook.1.7.1 PMC4781570 18050402

[42] Ciani L, Salinas PC (2005) WNTs in the vertebrate nervous system: from patterning to neuronal connectivity. Nat Rev Neurosci 6: 351–62. Available at: http://www.ncbi.nlm.nih.gov/pubmed/15832199. Accessed December 11, 2013. 15832199 10.1038/nrn1665

[43] Salinas PC, Zou Y (2008) Wnt signaling in neural circuit assembly. Annu Rev Neurosci 31: 339–58. Available at: http://www.ncbi.nlm.nih.gov/pubmed/18558859. Accessed January 2, 2014. 18558859 10.1146/annurev.neuro.31.060407.125649

[44] Lucas FR, Salinas PC (1997) WNT-7a induces axonal remodeling and increases synapsin I levels in cerebellar neurons. Dev Biol 192: 31–44. Available at: http://www.ncbi.nlm.nih.gov/pubmed/9405095. 9405095 10.1006/dbio.1997.8734

[45] Hall AC, Lucas FR, Salinas PC (2000) Axonal Remodeling and Synaptic Differentiation in the Cerebellum Is Regulated by WNT-7a Signaling. Cell 100: 525–535. Available at: http://linkinghub.elsevier.com/retrieve/pii/S0092867400806893. Accessed February 20, 2014. 10721990 10.1016/s0092-8674(00)80689-3

[46] Heisenberg CP, Tada M, Rauch GJ, Saúde L, Concha ML, et al (2000) Silberblick/Wnt11 mediates convergent extension movements during zebrafish gastrulation. Nature 405:76–81. 10811221 10.1038/35011068

[47] Schmitt AM, Shi J, Wolf AM, Lu CC, King LA, et al (2006) Wnt-Ryk signalling mediates medial-lateral retinotectal topographic mapping. Nature 439: 31–7. Available at: http://www.ncbi.nlm.nih.gov/pubmed/16280981. Accessed December 23, 2013. 16280981 10.1038/nature04334

[48] Packard M, Koo ES, Gorczyca M, Sharpe J, Cumberledge S, et al (2002) The Drosophila Wnt, wingless, provides an essential signal for pre- and postsynaptic differentiation. Cell 111: 319–30. Available at: http://www.pubmedcentral.nih.gov/articlerender.fcgi?artid=3499980&tool=pmcentrez&rendertype=abstract. 12419243 10.1016/s0092-8674(02)01047-4 PMC3499980

[49] Yoshikawa S, McKinnon RD, Kokel M, Thomas JB (2003) Wnt-mediated axon guidance via the Drosophila Derailed receptor. Nature 422: 583–8. Available at: http://www.ncbi.nlm.nih.gov/pubmed/12660735. 12660735 10.1038/nature01522

[50] Yu X, Malenka RC (2003) Beta-catenin is critical for dendritic morphogenesis. Nat Neurosci 6: 1169–77. Available at: http://www.ncbi.nlm.nih.gov/pubmed/14528308. Accessed January 9, 2014. 14528308 10.1038/nn1132

[51] Rosso SB, Sussman D, Wynshaw-Boris A, Salinas PC (2005) Wnt signaling through Dishevelled, Rac and JNK regulates dendritic development. Nat Neurosci 8: 34–42. Available at: http://www.ncbi.nlm.nih.gov/pubmed/15608632. Accessed December 30, 2013. 15608632 10.1038/nn1374

[52] Hilliard MA, Bargmann CI (2006) Wnt signals and frizzled activity orient anterior-posterior axon outgrowth in C. elegans. Dev Cell 10: 379–90. Available at: http://www.ncbi.nlm.nih.gov/pubmed/16516840. Accessed December 12, 2013. 16516840 10.1016/j.devcel.2006.01.013

[53] Pan CL, Howell JE, Clark SG, Hilliard M, Cordes S, et al (2006) Multiple Wnts and frizzled receptors regulate anteriorly directed cell and growth cone migrations in Caenorhabditis elegans. Dev Cell 10: 367–77. Available at: http://www.ncbi.nlm.nih.gov/pubmed/16516839. Accessed December 12, 2013. 16516839 10.1016/j.devcel.2006.02.010

[54] Goldstein B, Takeshita H, Mizumoto K, Sawa H (2006) Wnt signals can function as positional cues in establishing cell polarity. Dev Cell 10: 391–6. Available at: http://www.pubmedcentral.nih.gov/articlerender.fcgi?artid=2221774&tool=pmcentrez&rendertype=abstract. Accessed January 5, 2014. 16516841 10.1016/j.devcel.2005.12.016 PMC2221774

[55] Zinovyeva AY, Yamamoto Y, Sawa H, Forrester WC (2008) Complex network of Wnt signaling regulates neuronal migrations during Caenorhabditis elegans development. Genetics 179: 1357–71. Available at: http://www.pubmedcentral.nih.gov/articlerender.fcgi?artid=2475739&tool=pmcentrez&rendertype=abstract. Accessed December 17, 2013. 18622031 10.1534/genetics.108.090290 PMC2475739

[56] Klassen MP, Shen K (2007) Wnt signaling positions neuromuscular connectivity by inhibiting synapse formation in C. elegans. Cell 130: 704–16. Available at: http://www.ncbi.nlm.nih.gov/pubmed/17719547. Accessed December 15, 2013. 17719547 10.1016/j.cell.2007.06.046

[57] Farías GG, Vallés AS, Colombres M, Godoy JA, Toledo EM, et al (2007) Wnt-7a induces presynaptic colocalization of alpha 7-nicotinic acetylcholine receptors and adenomatous polyposis coli in hippocampal neurons. J Neurosci 27: 5313–25. Available at: http://www.ncbi.nlm.nih.gov/pubmed/17507554. Accessed January 9, 2014. 17507554 10.1523/JNEUROSCI.3934-06.2007 PMC6672358

[58] Jensen M, Hoerndli FJ, Brockie PJ, Wang R, Johnson E, et al (2012) Wnt signaling regulates acetylcholine receptor translocation and synaptic plasticity in the adult nervous system. Cell 149: 173–87. Available at: http://www.pubmedcentral.nih.gov/articlerender.fcgi?artid=3375111&tool=pmcentrez&rendertype=abstract. Accessed January 9, 2014. 22464329 10.1016/j.cell.2011.12.038 PMC3375111

[59] Mochizuki Y, Majerus PW (2003) Characterization of myotubularin-related protein 7 and its binding partner, myotubularin-related protein 9. Proc Natl Acad Sci U S A 100: 9768–73. Available at: http://www.pubmedcentral.nih.gov/articlerender.fcgi?artid=187840&tool=pmcentrez&rendertype=abstract. 12890864 10.1073/pnas.1333958100 PMC187840

[60] Mizumoto K, Shen K (2013) Two Wnts instruct topographic synaptic innervation in C. elegans. Cell Rep 5: 389–96. Available at: http://www.ncbi.nlm.nih.gov/pubmed/24139806. Accessed January 10, 2014. 24139806 10.1016/j.celrep.2013.09.011 PMC3898691

[61] Thorpe CJ, Schlesinger A, Carter JC, Bowerman B (1997) Wnt Signaling Polarizes an Early C. elegans Blastomere to Distinguish Endoderm from Mesoderm. Cell 90: 695–705. Available at: http://www.sciencedirect.com/science/article/pii/S0092867400805309. Accessed January 10, 2014. 9288749 10.1016/s0092-8674(00)80530-9

[62] Bolis A, Zordan P, Coviello S, Bolino A (2007) Myotubularin-Related (MTMR) Phospholipid Phosphatase Proteins in the Peripheral Nervous System. Mol Neurobiol 35: 308–316. Available at: http://link.springer.com/10.1007/s12035-007-0031-0. Accessed January 8, 2014. 17917119 10.1007/s12035-007-0031-0

[63] Prasad BC, Clark SG (2006) Wnt signaling establishes anteroposterior neuronal polarity and requires retromer in C. elegans. Development 133: 1757–66. Available at: http://www.ncbi.nlm.nih.gov/pubmed/16571624. Accessed December 12, 2013. 16571624 10.1242/dev.02357

[64] Baulac S, Gourfinkel-An I, Couarch P, Depienne C, Kaminska A, et al (2008) A novel locus for generalized epilepsy with febrile seizures plus in French families. Arch Neurol 65: 943–51. Available at: http://www.ncbi.nlm.nih.gov/pubmed/18625863. 18625863 10.1001/archneur.65.7.943

[65] Sanchez-Juan P, Bishop MT, Aulchenko YS, Brandel JP, Rivadeneira F, et al (2012) Genome-wide study links MTMR7 gene to variant Creutzfeldt-Jakob risk. Neurobiol Aging 33: 1487.e21–8. Available at: http://www.ncbi.nlm.nih.gov/pubmed/22137330. Accessed January 8, 2014. 10.1016/j.neurobiolaging.2011.10.011 22137330

[66] Mizumoto K, Shen K (2013) Interaxonal Interaction Defines Tiled Presynaptic Innervation in C. elegans. Neuron 77: 655–666. Available at: http://www.sciencedirect.com/science/article/pii/S0896627313000378. Accessed January 20, 2014. 23439119 10.1016/j.neuron.2012.12.031 PMC3846605

[67] Mello C, Fire A (1995) DNA transformation. Methods Cell Biol 48:451–82. 8531738

