# Supplementary figures and images for: MTM-6, a Phosphoinositide Phosphatase, is Required to Promote Synapse Formation in Caenorhabditis elegans
Source: PLoS One. 2014 Dec 5;9(12):e114501. doi: 10.1371/journal.pone.0114501 (PMC4257696; doi:10.1371/journal.pone.0114501)

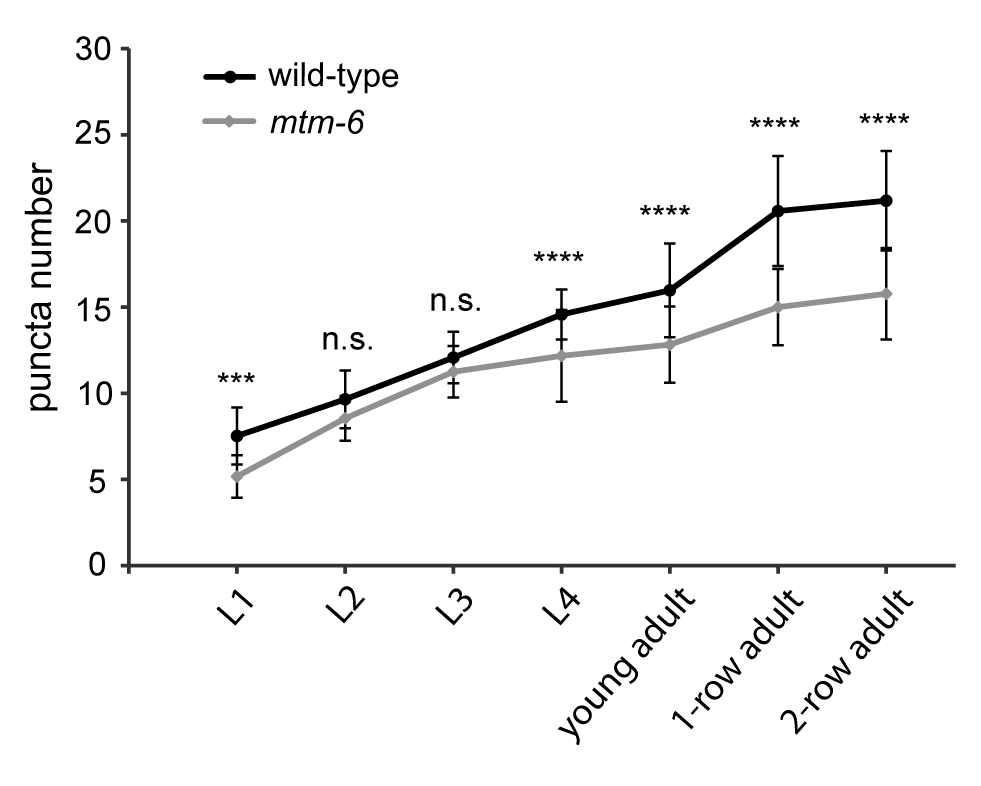

Supplement: Figure S1 — The mtm-6 phenotype is present at multiple stages of development. Puncta number for SNB-1::YFP were counted for wild-type and mtm-6 (ok330) animals at L1-L4, young adult, 1-row adult and 2-row adult. n = 40, *** is p<0.001, **** is p<0.0001, n.s. is not significant, and error bars are SD. (TIF) [file pone.0114501.s001.tif]

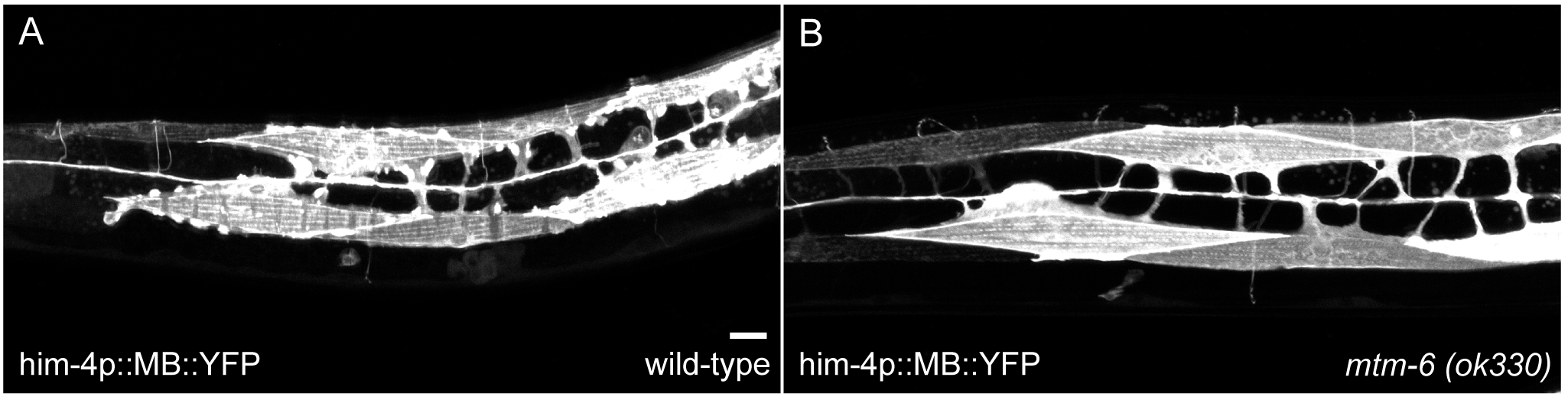

Supplement: Figure S2 — mtm-6 mutant animals demonstrate largely normal muscle structure. Muscle arms and muscle structure appear grossly normal when mtm-6 mutants (B) are compared with wild-type animals (A). (TIF) [file pone.0114501.s002.tif]

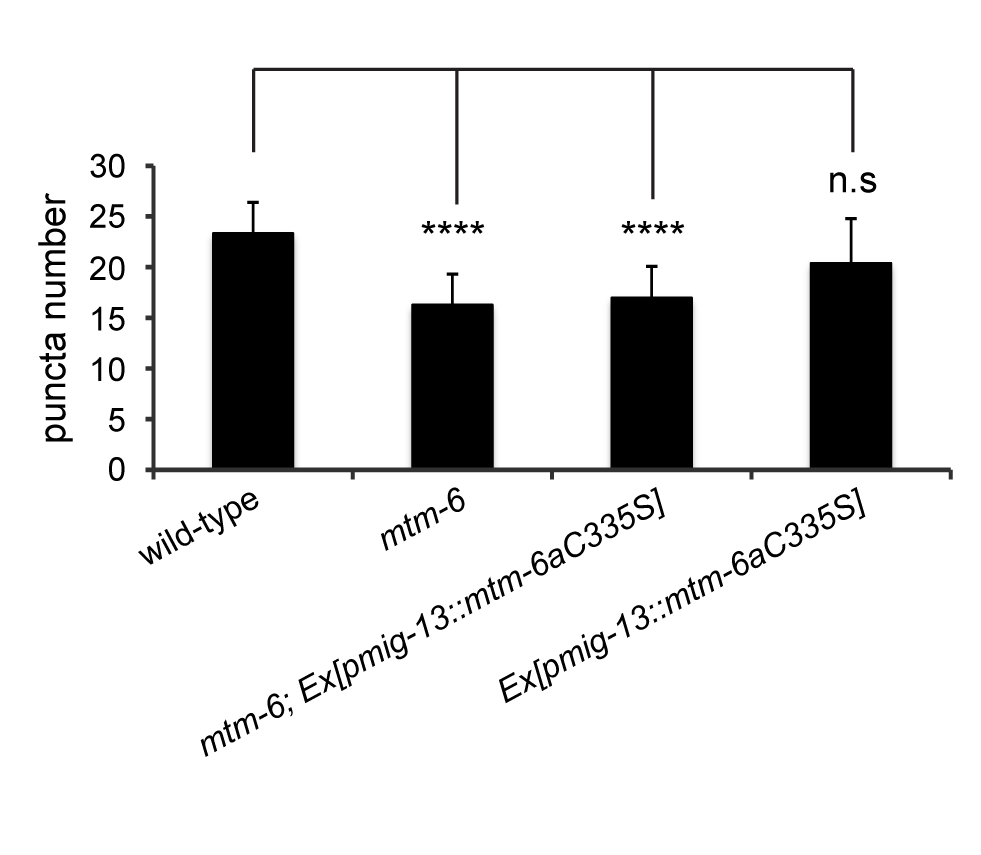

Supplement: Figure S3 — MTM-6 requires its phosphatase domain to maintain proper synapse number. A construct of mtm-6a containing a point mutation in the phosphatase domain failed to rescue the mtm-6 mutant. The construct had no affect on wild-type synapses. n>40, ****p<0.0001, n.s is not significant, and error bars are SD. (TIF) [file pone.0114501.s003.tif]

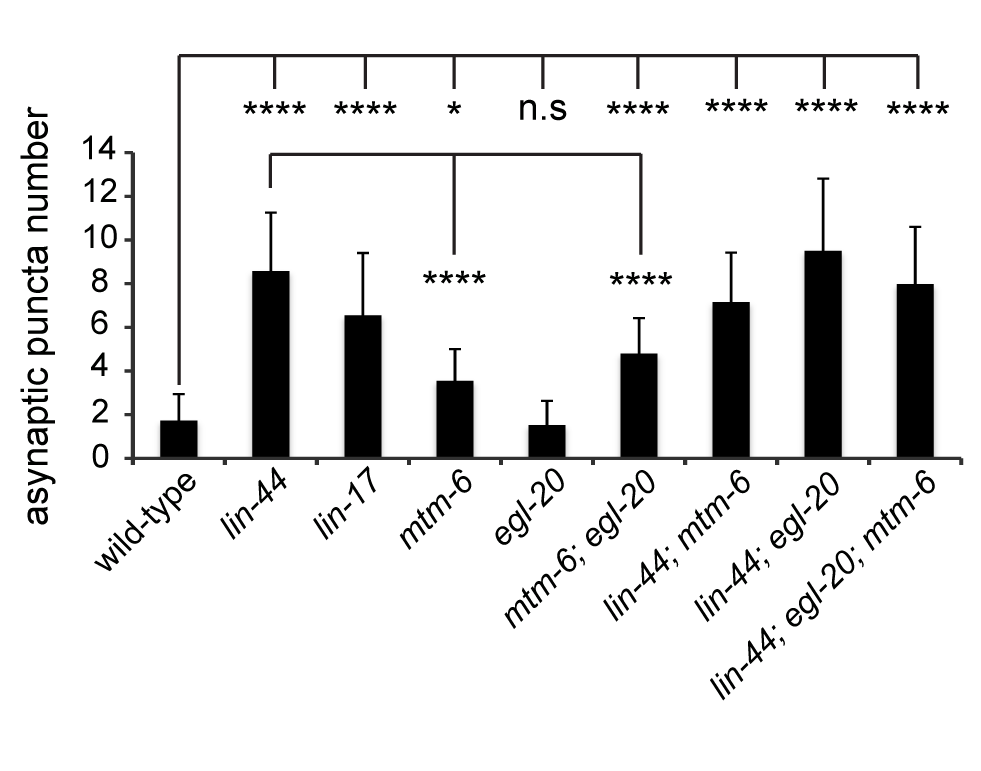

Supplement: Figure S4 — mtm-6 and mtm-6; egl-20 have a subtle asynaptic phenotype. Quantification of the aysynaptic shift phenotype in mtm-6 and mtm-6; egl-20 mutants compared to other Wnt mutants. n = 40, * is p<0.05, **** is p<0.0001, n.s is not significant, and error bars are SD. (TIF) [file pone.0114501.s004.tif]

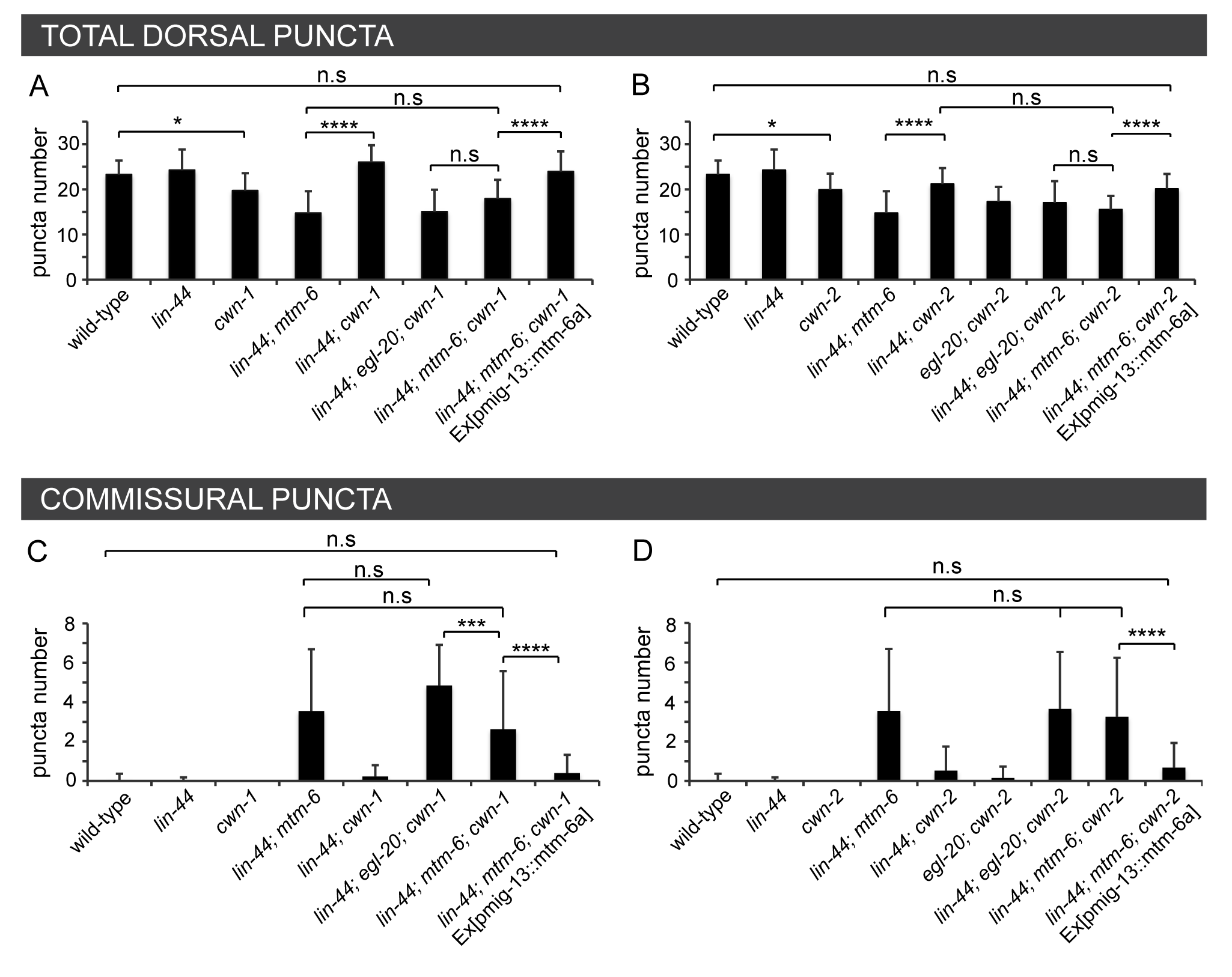

Supplement: Figure S5 — cwn-1 and cwn-2 mutants do not have the same pattern of Wnt enhancement as mtm-6 . Quantification and rescue data for different wnt mutants with cwn-1(ok546) (A and C) and cwn-2(ok895) (B and D). GFP::RAB-3 puncta were counted in the dorsal region (A and B) and the commissural region (C and D). n = 40,* is p<0.05, *** is p<0.001, **** is p<0.0001, n.s. is not significant, and error bars are SD. (TIF) [file pone.0114501.s005.tif]

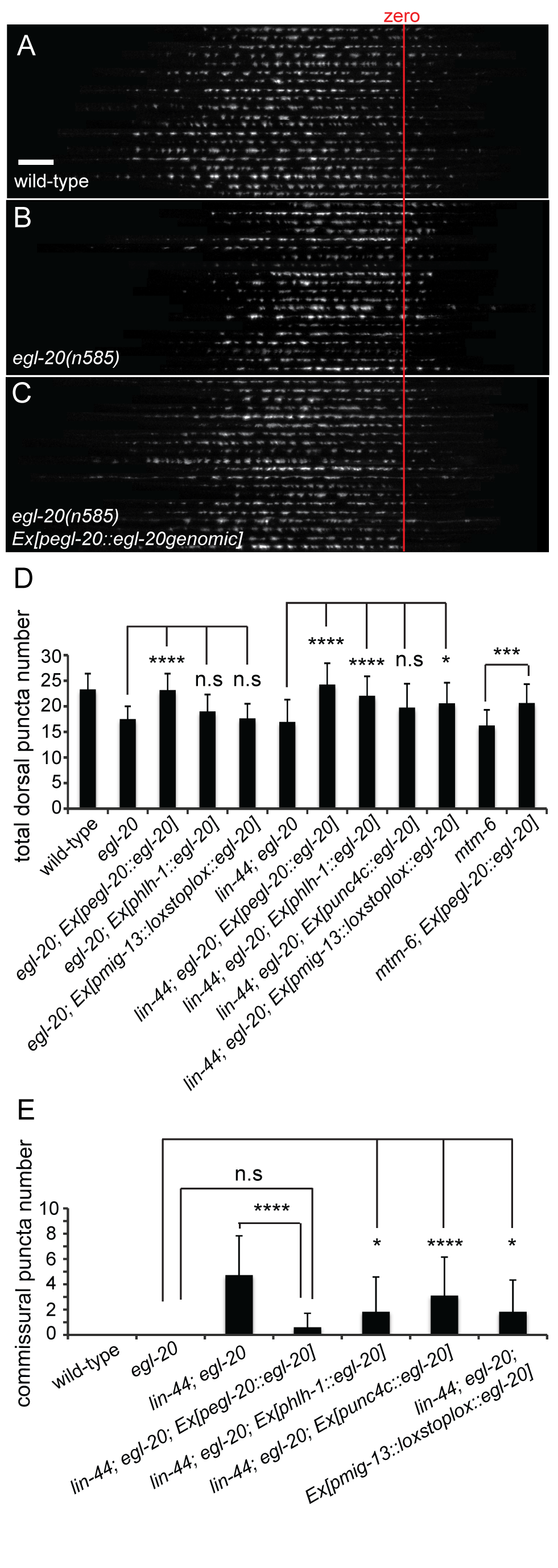

Supplement: Figure S6 — Egl-20 is not effectively rescued from neurons. Image montages of confocal images of GFP::RAB-3 puncta were collected for (A) wild-type, (B) egl-20(n585), (C) egl-20(n585); Ex[pegl-20::egl-20]. (D) Quantification of rescue for the total dorsal puncta phenotype of egl-20(n585) and lin-44(n1792); egl-20(n585). (E) Quantification of rescue for the commissural phenotype of egl-20(n585) and lin-44(n1792); egl-20(n585). n = 40,* is p<0.01, *** is p<0.001, **** is p<0.0001, n.s. is not significant, and error bars are SD. (TIF) [file pone.0114501.s006.tif]
